# “Fluctuograms” Reveal the Intermittent Intra-Protein Communication in Subtilisin Carlsberg and Correlate Mechanical Coupling with Co-Evolution

**DOI:** 10.1371/journal.pcbi.1002023

**Published:** 2011-03-24

**Authors:** Jordi Silvestre-Ryan, Yuchun Lin, Jhih-Wei Chu

**Affiliations:** 1 Department of Bioengineering, University of California, Berkeley, Berkeley, California, United States of America; 2 Department of Chemical and Biomolecular Engineering, University of California, Berkeley, Berkeley, California, United States of America; National Cancer Institute, United States of America and Tel Aviv University, Israel

## Abstract

The mechanism of intra-protein communication and allosteric coupling is key to understanding the structure-property relationship of protein function. For subtilisin Carlsberg, the Ca^2+^-binding loop is distal to substrate-binding and active sites, yet the serine protease function depends on Ca^2+^ binding. The atomic molecular dynamics (MD) simulations of apo and Ca^2+^-bound subtilisin show similar structures and there is no direct evidence that subtilisin has alternative conformations. To model the intra-protein communication due to Ca^2+^ binding, we transform the sequential segments of an atomic MD trajectory into separate elastic network models to represent anharmonicity and nonlinearity effectively as the temporal and spatial variation of the mechanical coupling network. In analogy to the spectrogram of sound waves, this transformation is termed the “fluctuogram” of protein dynamics. We illustrate that the Ca^2+^-bound and apo states of subtilisin have different fluctuograms and that intra-protein communication proceeds intermittently both in space and in time. We found that residues with large mechanical coupling variation due to Ca^2+^ binding correlate with the reported mutation sites selected by directed evolution for improving the stability of subtilisin and its activity in a non-aqueous environment. Furthermore, we utilize the fluctuograms calculated from MD to capture the highly correlated residues in a multiple sequence alignment. We show that in addition to the magnitude, the variance of coupling strength is also an indicative property for the sequence correlation observed in a statistical coupling analysis. The results of this work illustrate that the mechanical coupling networks calculated from atomic details can be used to correlate with functionally important mutation sites and co-evolution.

## Author Summary

A hallmark of protein molecules is their machine-like behaviors while carrying out biological functions. At the molecular level, molecular signals such as binding a metal ion at an action site can cause long-range effects and alter protein function. Such phenomena are often referred to as intra-protein communication or allosteric coupling. Elucidating the underlying mechanisms could lead to novel discovery of molecular modulators to regulate protein function in a more specific and effective manner. A long-standing puzzle is the roles of the anharmonicity and nonlinearity in protein dynamics. To incorporate these characters in modeling intra-protein communication, we devise a “fluctuogram” analysis to record the choreography of allosteric coupling in an atomic molecular dynamics simulation. We show that fluctuogram analysis can bridge the results of physics-based simulation and sequence alignment in bioinformatics by capturing the residues that exhibit high correlation in a multiple sequence alignment. We also show that the fluctuograms calculated from atomic details have the potential to be applied as a tool to select mutation sites for modulating protein function.

## Introduction

During protein dynamics, the temporal and spatial couplings between amino acids are governed by the atomic details encoded in the sequence and protein's environment. A critical outcome is that ligand binding, chemical modification, and changes in solvent conditions not only alter structures and thermal motions locally: molecular signals can propagate through the protein matrix and affect the properties of distal sites [1], [2], [3]. For subtilisin Carlsberg, the Ca^2+^-binding loop in proximity to the N-terminal is distant to the substrate-binding and active sites, yet the protease function and stability depends on Ca^2+^ binding [4], [5], [6], [7]. Allosteric coupling is a ubiquitous mechanism by which protein functions are regulated and coordinated in the cell [8], [9], [10]. Mechanistic understanding at the molecular level, though, is still under development.

The classical induced-fit and population shift models highlight two essential features of intra-protein communication: the mechanical coupling (interaction energetics) between amino acids and the ensemble distribution of protein structures [1], [2], [3]. According to the induced-fit theory, molecular signals at a site induce local conformational changes and affect residues in the next layer via mechanical coupling [1]. The propagation of molecular signals may thus follow a sequential (stepwise) path [11], [12], [13] and pathways of allosteric coupling may be defined based on the contacted amino acids observed in a protein structure [14], [15], [16], [17], [18]. The population shift model emphasizes that the ensemble distribution of protein structures depends on ligand binding or other forms of molecular signals [2], and the equilibria between pre-existing conformations would shift as a result [11], [12], [13]. The response of structural distribution is often non-linear, leading to properties such as cooperative binding. It has been shown in many examples that the population shift model can be used to predict the thermodynamics of allosteric coupling and protein stability [19], [20].

The ensemble distribution of protein conformation can be represented by the potential of mean forces (PMF) of the relevant degrees of freedom used for structural description, such as the positions of all heavy atoms and polar hydrogen. Other degrees of freedom are considered averaged out according to statistical mechanics [21], [22]. The mean forces not only reflect the mechanical coupling network in protein structure, their integration also determines the ensemble distribution of protein conformation. Therefore, variation in the mechanical coupling network of protein structure due to molecular signals is linked to allosteric coupling as well as the concomitant population shift.

Subtilisin Carlsberg is a serine protease widely used in industry and protein engineering studies [4], [5], [6], [7]. Similar to numerous enzymes and signaling proteins, the functioning of subtilisin is regulated by Ca^2+^. Subtilisin has a strong Ca^2+^-binding site with a dissociation constant of ∼100 nM and Ca^2+^ exhibits significant effects on stability and folding kinetics [23], [24]. The fold of an engineered construct without the Ca^2+^-binding loop is very close to that of native subtilisin [25], and there is no direct evidence of alternative structure. Ca^2+^-mediated intra-protein communication in subtilisin may thus proceed via local variation in the mechanical coupling network.

To test this hypothesis, we consider the anharmonicity and nonlinearity of protein dynamics in an effective manner. First, we recognize that the ensemble distribution of protein structures is determined by the mechanical coupling between amino acids, and shifts in the population of protein structures would reflect in the variation of mechanical coupling network. As the PMF of protein structure is extremely complex, modeling usually employs simplified basis functions [21], [22]. Here, we use an elastic network model (ENM) [26], [27] to approximate the distribution of protein structures. As the structural distribution corresponding to an ENM is determined by model parameters, we adjust the lengths and force constants of elastic bonds to match the statistics of structural fluctuations collected in a molecular dynamics (MD) simulation with explicit solvent [28]. The atomic details encoded in the sequence and protein's environment are thus reflected in the values of model parameters. The simplicity of harmonic potentials allows for the development of robust computational methods such as fluctuation matching for inverting structural fluctuations into force constants [28], [29], which we employ for all of the calculations performed in this work.

To effectively represent the anharmonicity and nonlinearity in protein dynamics, we compute separate ENM's from the sequential segments of a long MD trajectory to follow the time evolution of the mechanical coupling network in subtilisin. In analogy to the spectrogram of sound waves (temporal variation of spectral density) widely used in the fields of linguistics and speech recognition [30], we refer to the temporal variation of the mechanical coupling network as “fluctuogram”, which records the choreography of protein dynamics.

We computed the fluctuograms of Ca^2+^-bound and apo subtilisin from 100 ns all-atom MD trajectories in explicit water. The calculated fluctuograms demonstrate that intra-protein communication proceeds intermittently both in space and in time. We found that residues with large mechanical coupling variation due to Ca^2+^ binding significantly overlap with the gain-of-function mutation sites reported in the directed evolution studies that aim to enhance the stability and activity of subtilisin by random mutations and screening [31], [32], . Furthermore, we utilize the fluctuograms calculated from atomic MD simulation to capture the highly correlated residues in a multiple sequence alignment. In addition to the strength of mechanical coupling, we show that the variance of coupling strength is also an indicative property for the high sequence correlation observed in a statistical coupling analysis [37], [38]. Overall, our results illustrate that the mechanical coupling networks and fluctuograms calculated from atomic details can be used to correlate with functionally important mutation sites and co-evolution.

## Results

**Figure 1:**
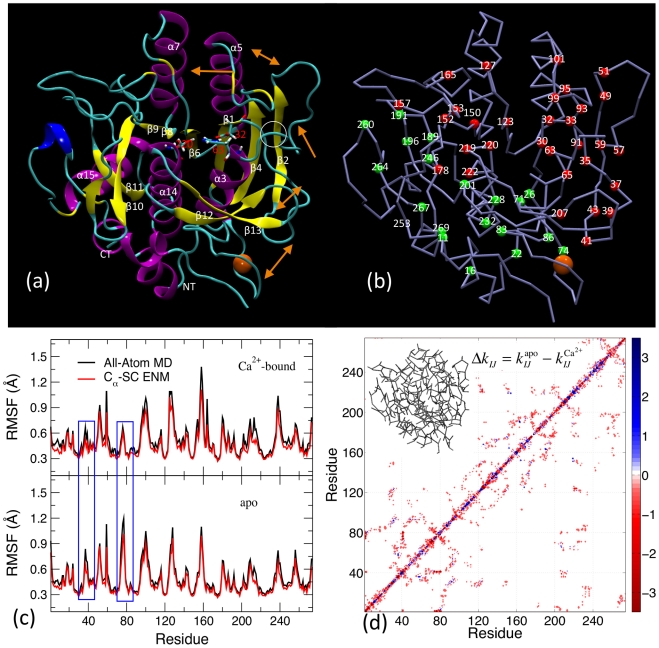
The structure and mechanical coupling network of subtilisin. (a) A ribbon representation of the X-ray structure of subtilisin (PDB ID: 1OYV). The bound Ca^2+^ is shown in ball. The secondary structural elements are labeled and the residues of the catalytic triad are listed. A sequential conformational change that represents a pathway of intra-protein communication induced by Ca^2+^ binding is shown via orange arrows. (b) Residues exhibit significant mechanical coupling in subtilisin. Residues cover the upper-right half are colored red and those cover the lower-left are colored green. (c) The root of mean square fluctuation (RMSF) of C_α_ atoms in Å calculated from the first 4 ns of Ca^2+^-bound (top) and apo (down) simulations. (d) Contour plot of the difference in inter-residue force constant (kcal/mol/Å^2^) between Ca^2+^-bound and apo simulations. Force constants are calculated from the first 4 ns of Ca^2+^-bound and apo simulations.

The native structure of subtilisin shown in Figure 1(a) has 17 segments of helices and sheets connected by loops and turns. Subtilisin contains several commonly encountered right-handed βαβ motifs and one rarely encountered left-handed βαβ motif (β2-α3-β4), for which the β1–β2 loop (Asp32-Asp41) and the β2-α3 loop (Ser49-His63) cross each other as circled in Figure 1(a). We name the loops and turns of subtilisin based on the structural elements that they connect; i.e., the β1–β2 loop connects β1 and β2. In the Ca^2+^-bound and apo trajectories of subtilisin, the time evolution of C_α_ root-mean-square differences (RMSDs) show that both Ca^2+^-bound and apo subtilisin remained close to the reference X-ray structure with RMSDs ∼1.5 Å (Figure S1).

We also calculate the RMSFs (root-mean-square fluctuation) of C_α_ atoms to quantify their flexibility; values from the first 4 ns trajectory are shown in Figure 1(c). Residues in loops and turns are more flexible than those in rigid secondary structures as expected. A clear feature is that apo subtilisin has higher RMSF's in the Ca^2+^-binding loop (Val71-Leu83) and around Asp41 (highlights in Figure 1(c)). The negatively charged Asp41 in the β1–β2 loop (Asp32-Asp41) loop coordinates with Ca^2+^ if present. The RMSF's predicted via the C_α_-SC-ENM (SC≡sidechain) are also shown in Figure 1(c) to illustrate that the RMSF's observed in all-atom MD are preserved at the coarse-gain scale by using fluctuation-matched force constants.

To capture the anharmonicity and nonlinearity sampled in all-atom MD simulations, in each of the sequential time windows of a user-specified size, we calculate the bond lengths of a C_α_-SC-ENM as mean distances and the force constants by fluctuation matching [28]. In analogy to the spectrogram of sound waves, the temporal evolution of the C_α_-SC-ENM is termed the “fluctuogram”, which records the choreography of protein dynamics. The window size 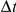 is an adjustable parameter, which specifies the timescale with which the Hamiltonian of a C_α_-SC-ENM is used to approximate the structural fluctuations of subtilisin. A small 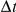 gives high time resolution but force constants are determined with a fewer number of configurations. A larger 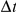 gives lower time resolution but the force constants are determined with more configurations. Another consideration is that C_α_-SC-ENM becomes less representative for configurations sampled in a longer MD segment, and we limit 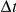 to a few ns for fluctuogram calculations. Over 100 ns atomic trajectories, we employ a window size of 4 ns. Fluctuograms with 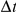 = 2 ns or 10 ns are qualitatively similar (results not shown). We also overlap the sequential time windows by 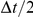 to better resolve the transitions around the timescale of 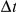. In the following, we characterize mechanical coupling variation and mechanisms of intra-protein communication via fluctuograms.

### The mechanical coupling network in subtilisin

The mechanical coupling between residues *I* and *J* is represented by 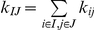, where *i* and *j* are the indices for CG sites. Fluctuation matching determines the force constants from the statistics of inter-site distances [28]. Differences in 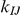
's between Ca^2+^-bound and apo simulations for the first 4 ns are shown in Figure 1. Many 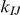
's in apo subtilisin are lower than those in the Ca^2+^-bound state, even though the structures are close to the initial X-ray structure and to each other.

The off-diagonal features in Figure 1(d) are due to tertiary contacts, and a wide range of the values of force constants are observed. The strong electrostatic coupling between Asp41 and Ca^2+^ (Figure 1(b)) results in a very large force constant of 133 kcal/mol/Å^2^, while the His39-Thr207 coupling in Ca^2+^-bound subtilisin has a force constant of 6.5 kcal/mol/Å^2^. Force constants between *I*-*I*+4 residue pairs in α helices are 2–7 kcal/mol/Å^2^. Therefore, a cutoff of 2.5 kcal/mol/Å^2^ is used to assign whether a residue pair with sequence difference larger than three has significant mechanical coupling. The force constants of covalent linkages along the peptide backbone (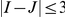) are significantly larger than those of 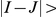
3.

Representative residues with many instances of significant mechanical coupling (*k_IJ_*>2.5 kcal/mol/Å^2^) and larger sequence separation (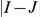
>3) are shown in Figure 1. Following residue pairs with significant mechanical coupling, the Ca^2+^ binding loop (Val71-Leu83) can be linked to distal regions in subtilisin. This result is based on the statistics of structural fluctuations via fluctuation matching and affirms that intra-protein communication can occur through the mechanical coupling network in subtilisin. An important residue is Asp41, which coordinates with Ca^2+^ if present. Asp41 locates at the C-terminal end of the β1–β2 loop (Asp32-Asp41), and Asp32 at the other end is one of the three catalytic triad residues (Asp32, His63, and Ser220). As *k*_33,95_ is significant, the Ca^2+^ binding loop can be linked to Leu95 from Asp41 via Thr33 (Figure 1(b)). The junction at Asp32 in the β1–β2 loop is mechanically coupled to the site around His63, a triad residues located in the β2-α3 loop (Ser49-His63), which also couples with the catalytic Ser220 in the α14 helix (Thr219-Lys236). Molecular signals at Ca^2+^-binding loop can thus propagate to the active site from Asp41 through residue pairs having significant mechanical coupling. In establishing this link, we also apply the fact that amino acids close in sequence are mechanically coupled through the backbone [39]. Tertiary contacts with strong mechanical coupling provide shortcuts to residues with larger sequence separation. In Figure 1(b), residues with significant mechanical coupling that cover the upper-right half of subtilisin are colored in red.

In addition to Asp41, the terminals of the Ca^2+^-binding loop, Val71 and Leu83, are mechanically coupled to the surrounding residues. Originated from the ends of the Ca^2+^-binding loop, the residue pairs with significant mechanical coupling that cover the lower-left half of subtilisin are colored in green in Figure 1(b). The grouping of red and green residues is a structure-based categorization, and does not grant their independence. In fact, red and green residues meet at the α14 helix (Ser220-Lys236) and the β8–β9 loop (Gly153-Asp171) and have multiple instances of direct mechanical coupling.

### Ca^2+^-binding modulates the mechanical coupling network in subtilisin

**Figure 2:**
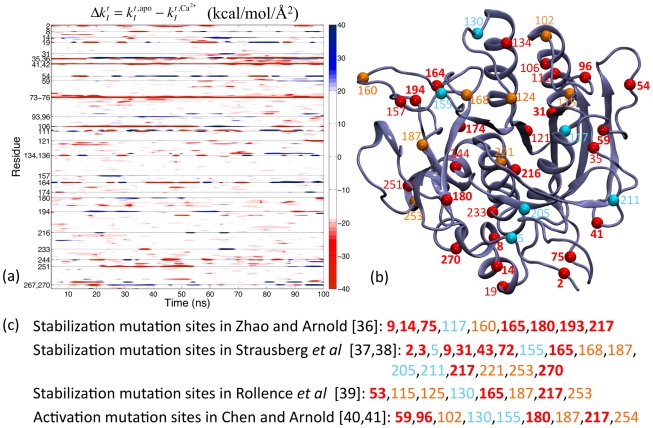
Mechanical coupling variation of subtilisin due to Ca^2+^ binding. (a) Differences in the force constant of each residue between the Ca^2+^-bound and apo simulations of subtilisin as a function of time, 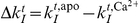 (kcal/mol/Å^2^). Residues with large mechanical coupling variation are highlighted in the y-axis. See text for the definition of 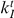
's. (b) The location of the residues highlighted in (a) and (c). Residues specified by *red* fonts: residues have large mechanical coupling variation to Ca^2+^ binding, i.e. the average of 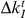
's is larger than 20 kcal/mol/Å^2^. Residues specified by *red* and *boldfaced* fonts: residues with large mechanical coupling variation and cover the mutation sites listed in (c) to within ±1. Residues specified by *red* and *not boldfaced* fonts: residues with large mechanical coupling variation but are not within ±1 of any of the mutation sites listed in (c). Residues specified by *orange* fonts: mutation sites listed in (c) with significant but not large mechanical coupling variation due to Ca^2+^ binding, i.e., the time average of 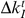
's is in between 10–20 kcal/mol/Å^2^. Residues specified by *light blue* fonts: mutation sites listed in (c) with medium or weak mechanical coupling variation, i.e., the time average of 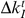
's is less than 10 kcal/mol/Å^2^. (c) Mutation sites reported in protein engineering literature that can enhance the stability of subtilisin and the activity in a non-aqueous solvent. The residues are colored and boldfaced according to the criteria described in (b).

The force constants of elastic bonds provide a direct measure of the mechanical coupling between amino acids. From the atomic configurations sampled in time window *t*, the force constant between *ij* sites, 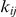, is calculated by fluctuation matching [28]. The mechanical coupling between residues *I* and *J* is determined as 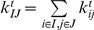. The mechanical coupling associated with residue *I* is then calculated as 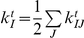 and the difference in 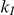 between Ca^2+^-bound and apo simulations in a time window is 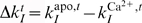. The profiles of 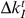 are shown in Figure 2(a). It can be seen that Ca^2+^-mediated interactions make certain regions in apo subtilisin becoming more flexible and others less. The compensatory balance in mechanical coupling variation is discussed in detail in Figure S2 and Text S1.

Subtilisin sites with large mechanical coupling variation often occur at loops and the connecting regions of rigid secondary structures, Figure 2(a,b). These sites, however, are highly specific and not all flexible regions have large mechanical coupling variation. The 25 most affected residues in subtilisin (within top 10%) due to Ca^2+^ binding (the time average of 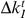
's>20 kcal/mol/Å^2^) are listed in Figure 2(a), and their spatial locations are shown in Figure 2(b) in red. As an example, around Asp75 at the edge of the Ca^2+^-binding loop and Asp41 that coordinates Ca^2+^ if present, 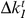 have large and negative values, indicating weaker mechanical coupling in apo subtilisin. The nearby N-terminal site (residue 2) shows a similar behavior. In addition to such anticipated results, it is clear from Figure 2(a,b) that Ca^2+^-binding causes mechanical coupling variation not only locally around the Ca^2+^-binding loop but also residues that are far away. Through the mechanical coupling network in subtilisin, the local molecular signal of Ca^2+^ binding propagates across the network and causes variation at distal sites.

Since the stability of subtilisin strongly depends on Ca^2+^, residues with large mechanical coupling variation between Ca^2+^-bound and apo simulations may be hot spots for modulating protein stability. To test this hypothesis, we compare the residues shown in red in Figure 2(a,b) with those identified by random mutations and screening to have positive effects on activity and stability. Since subtilisin has been used as a model system for methodology development in protein engineering [7], many mutation sites had been identified. For example, in converting subtilisin E to its thermophilic homolog via directed evolution, Zhao and Arnold found 9 mutation sites after screening ∼50,000 clones [31]. Mutations at these sites (Figure 2(c)) increase subtilisin lifetime at 60°C >200 times longer than that of the wild type [31]. Among the 9 sites identified by Zhao and Arnold, 7 (9, 14, 75, 165, 180, 193, 217) are covered to within ±1 in residue number by the 25 sites calculated from atomic MD simulations for having large mechanical coupling variation (Figure 2(a,b)). The specific amino acid type of a mutant residue is definitely a key in gaining function in directed evolution, but here we focus on comparing the location of mutation sites.

The 7 covered residues are listed as boldfaced fonts in red in Figure 2(b,c). On average, randomly picking 25 residues only covers 1–3 out of the 9 residues identified by directed evolution. 10,000 runs of random picking were performed to calculate the average and variance of covering the reported mutation sites; using 1000 runs gives quantitatively close results.

Among the 9 reported mutation sites, even though residue 160 is not covered, its calculated mechanical coupling variation is actually quite significant; the average of 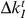
's is 15.4 kcal/mol/Å^2^. If the residues selected by directed evolution have significant but not large mechanical coupling variation, i.e., the time average of 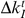
's is between 10–20 kcal/mol/Å^2^, they are colored orange in Figure 2(b,c). If the residues selected by directed evolution have medium or small mechanical coupling variation, i.e., the average of 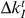
's is less than 10 kcal/mol/Å^2^, they are colored light blue in Figure 2(b,c). If the residues with large mechanical coupling variation (red) are not within ±1 of any of the reported mutation sites, they are labeled via a red, unboldfaced font in Figure 2(b).

Ca^2+^ binding is a molecular signal known to affect the stability of subtilisin. Atomic simulations and fluctuation matching reveal that it indeed has significant effects on the mechanical coupling network in subtilisin, Figure 2(a,b). The results of directed evolution in [31] suggest that most of the identified mutation sites that demonstrate stabilization effects have high susceptibility in mechanical coupling. In protein engineering, it is often observed that the mutant residues survived from random mutation locate at loops or connecting regions between rigid secondary structures, probably because the mutations therein are more tolerable [40]. In subtilisin, this trend is also observed but mutation sites in well-defined secondary structures are also identified as well, Figure 2(a,b).

Residues with large mechanical coupling variation also tend to locate at loops and connecting regions as shown in Figure 2(a), but only specific residues would satisfy a designated selection criterion such as the average of 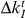
's is larger than 20 kcal/mol/Å^2^. The mechanical coupling calculated from MD simulations reflects the sequence-specific thermodynamic interactions between residues. The correlation between the stabilization mutation sites and the residues with large mechanical coupling variation suggests that having different thermodynamic interactions with the surrounding could be an indicative property for a residue to be an effective mutation site for protein engineering. To further test this theory, we compare simulation results with other protein engineering works.

The strong Ca^2+^-dependence of stability and folding kinetics of subtilisin makes its application as an industrial enzyme difficult; eliminating Ca^2+^ dependence has thus been a long-standing interest in subtilisin engineering. Removing the sequence of the Ca^2+^-binding loop in subtilisin BPN' has been shown to achieve this objective but at the expense of significantly reduced stability. Strausberg *et al* [32], [33] integrated the reported mutation sites of subtilisin variants and increased the stability (half-life at 75°C) of their Ca^2+^-free construct 15,000 folds by directed evolution. The 17 mutations sites that were involved in achieving this success are shown in Figure 2(c). Residue sites 9, 165, and 217 agree with the results in Zhao and Arnold [31], and residue 72 was selected instead of 75 after removing the Ca^2+^-binding loop. 9 of the stabilization mutation sites employed in Strausberg *et al* [32], [33] are covered by the residues with large mechanical coupling variation (red boldfaced); other 4 residues have weaker but significant mechanical coupling variation (orange), Figure 2(b,c). In a different protein engineering study by directed evolution, most of the stabilization mutation sites reported in Rollence *et al* [34] are also in agreement with those in Zhao and Arnold [31] and Strausberg *et al* [32], [33], and also are listed in Figure 2(c). In total, the 25 calculated residue sites with large mechanical coupling variation cover 14 of the 25 stabilization mutation sites reported in [31], [32], [33], [34] to within ±1. Randomly picking 25 residues can only cover 4–8 residues, supporting the theory that the susceptibility of mechanical coupling to functionally important signals such as Ca^2+^ binding is an indicative property for a residue to be an effective mutation site in protein engineering.

In addition to stability, the mechanical coupling network in protein structure also affects conformational flexibility and protein dynamics. It is thus expected that varying mechanical coupling network would also alter other functional properties such as substrate binding and activity. In applying subtilisin as an industrial enzyme, one desired property is the ability to function in non-aqueous environments. This property has been shown to relate to the flexibility and dynamics of protein conformation [41], [42], [43]. In enhancing the activity of subtilisin E in a solution with a high concentration of a polar organic solvent by directed evolution, Chen and Arnold had identified 9 mutation sites that increase the activity in 60% dimethylformamide to 256 times that of the wild type [35], [36]. These residue sites also shown in Figure 2(c). Residues 59, 96, and 102 are distinct and the other 6 are in the pool of the stabilization mutation sites reported in [31], [32], [33], [34]. Residues 96 and 102 are in the β4-α5 loop (93–104) that involves substrate binding; residue 59 is in the β2-α3 loop (49–63) that extends from His63 in the catalytic triad. Residue 59 and 96 have large (red boldfaces) and 102 has weaker but significant mechanical coupling variation (orange), Figure 2(b,c). The functional relevance of mechanical coupling variation is thus not limited to stability. Another residue with large mechanical coupling variation is 174, which had been shown to modulate the Ca^2+^ binding of subtilisin at the weaker binding site [44].

Out of the 28 mutation sites reported in [31], [32], [33], [34], [35], [36] that had been shown to enhance the stability and activity of subtilisin, 16 are covered to within ±1 by the 25 residues calculated to have large mechanical coupling variation, Figure 2(b,c); randomly picking 25 residues only covers 5–9 residues. The ratio of the number of captured mutation sites to the number of selected residues, 0.64, also far exceeds the corresponding values achieved via random picking, 0.3±0.09. These results indicate that the mechanical coupling networks calculated from atomic details can be used to correlate with the functionally important mutation sites selected by directed evolution.

Another feature in Figure 2(a) is that mechanical coupling variation is intermittent. In the following, we analyze the intrinsic intermittence in the dynamics of Ca^2+^-bound and apo subtilisin and explore its functional relevance.

### Intermittent conformational changes and mechanical coupling variation in subtilisin

The variation of 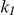 between consecutive time windows, 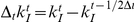, of apo and Ca^2+^-bound subtilisin (Figure S3) shows an intermittent pattern similar to that of 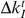
's in Figure 2(a). Intermittence in 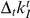 indicates that during protein dynamics, increases in mechanical coupling strength for a peptide segment do not last extensively long. As the segment enters a resting period, reduction in flexibility or mechanical coupling strength tend to follow, although further increases after the resting period are observed occasionally as well (Figure S3). Prominent features in 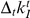
's thus alternate among different sites with time. This behavior illustrates that protein structural fluctuations are highly rectified. In the following, we first establish correspondences between conformational changes and mechanical coupling variation and characterize the pathways of intra-protein communication.

**Figure 3:**
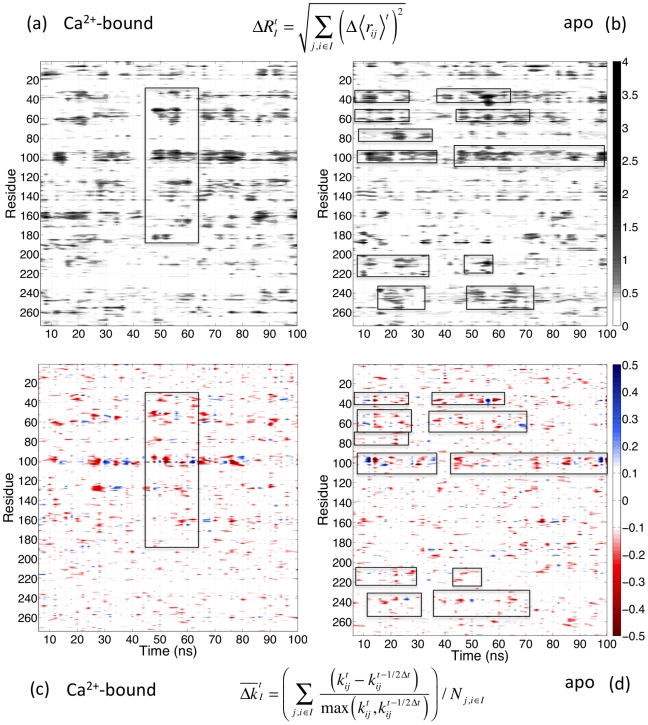
Changes in the local conformation and mechanical coupling of each residue in subtilisin between neighboring time windows. (a) Conformational changes in the Ca^2+^-bound simulation. (b) Conformational changes in the apo simulation. The change in inter-site distance in Å between two neighboring time windows, 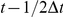 and *t*, is 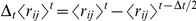 and the local conformational change of residue *I* is defined as 
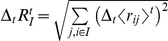
. Variation in the mechanical coupling of each residue between neighboring time windows for (c) the Ca^2+^ simulation and (d) the apo simulation. Mechanical coupling variation of residue *I* between two neighboring time windows, 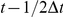 and *t*, is defined as 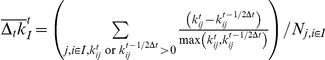. 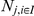 is the number of *ij* pairs associate with residue *I* and with at least one of 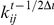 or 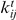 has positive value. The time window 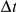 for calculating 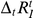
and 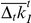 is 4 ns.

The change of a bond length in the mechanical coupling network between time windows is: 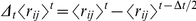. The overall conformational change of residue *I* is estimated by adding 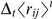
's together: 
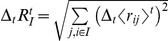
. To monitor conformational changes relevant to mechanical coupling, only bonds with non-zero 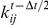 or 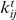 are involved in the sum. 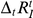
's for Ca^2+^-bound and apo simulations are shown in Figure 3(a) and Figure 3(b), respectively.

If a peptide segment in subtilisin underwent conformational changes over a period of time, 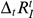
's of these residues shows up as a band. For regions with limited mobility, 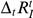
's are small. If mechanically coupled segments underwent correlated conformational changes, 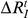 bands would appear together or close in time. In Ca^2+^-bound subtilisin, co-occurring 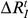 bands in β1–β2 loop (Asp32-Asp41), β2-α3 (Ser49-His63), β4-α5 (Lys93-Ser104), β6-α7 (Met123-Thr132), and β8–β9 (Gly153-Asp171) loops are clear in Figure 3(a), and a set of collective 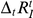 bands spanning ∼20 ns is highlighted as an example. This event corresponds to a sequentially collective conformational change with mechanically coupled residues; the details are shown in Figure S4.

Since the values of force constants for residue pairs close in sequence (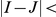
3) are much larger than those of tertiary contacts, variations of bare 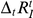
's (Figure S3) tend to under-represent the mechanical coupling variation between tertiary contacts and do not show a close correspondence with 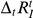
's. To establish a tighter connection between mechanical coupling variation and local conformational changes, a useful parameter is:
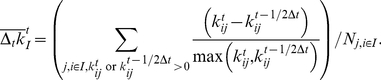
(1)In eq.(1), 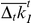 is the average of relative differences in force constants for the bonds that are connected to residue *I*. Only bonds with a non-zero 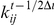 or 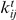 are considered; 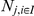 is the number of such *ij* pairs. 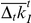
's of Ca^2+^-bound and apo simulations are shown in Figure 3(c) and Figure 3(d), respectively. Normalizing 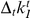 by 
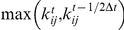
 in 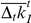 incorporates larger contributions from the tertiary contacts, and 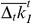
's thus follow 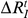
's more closely than 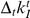
's. Furthermore, 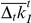
's vary between ±1 and provide a simple metric for estimating the extent of the mechanical coupling variation of residue *I*. Since 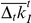
's closely follow the intermittent features of 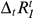
's in Figure 3, a tight connection between conformational change and mechanical coupling variation is established. Prominent 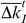
's can be observed right before, after, or around 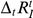 bands.

The fluctuograms shown in Figure 3 record the chorography of protein dynamics with a time window of 4 ns. The movies of the equilibrium structures of sequential C_α_-SC-ENM's further illustrate the intermittence of conformational changes and are provided in VideoS1 and VideoS2. Fluctuograms using 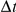 = 2 ns and 10 ns show qualitatively similar patterns (results not shown).

### Intra-protein communication due to Ca^2+^ binding

The fluctuogram of apo subtilisin (Figure 3(b,d)) records a choreography that the signal of removing Ca^2+^ propagates through the mechanical coupling network and affects active and substrate-binding sites that are 20–30 Å away. Such behavior is not seen in the fluctuograms of Ca^2+^-bound subtilisin (Figure 3(a,c)), which record a different pattern of choreography. Here, the apo fluctuograms are analyzed in detail; the analyses of Ca^2+^-bound fluctuograms are discussed in Figure S4 and Text S1.

In apo subtilisin, the absence of Ca^2+^ caused prominent bands in 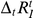 and 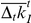 in the Ca^2+^-binding loop (Val71-Leu83) as highlighted in Figure 3(b,d). Since Asp41 in the β1–β2 loop (Asp32-Asp41) loop tightly coordinates with Ca^2+^ if present, the absence of Ca^2+^-mediated interactions affects the mechanical coupling of this loop, and the β1–β2 loop in apo subtilisin has larger intermittent bands, as highlighted in Figure 3(b,d). Despite that the force constants at this region show large differences between Ca^2+^-bound and apo subtilisin (Figure 2(a)), differences in intrinsic mechanic coupling variation are also clear. It is obvious from Figure 3(b,d) that 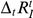 and 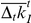 bands in the β2-α3 loop occur close in time with those in the β1–β2 loop: Ca^2+^-mediated changes continue to affect the β2-α3 loop through mechanical coupling.

Mechanical coupling also causes β1–β2 (Asp32-Asp41), β2-α3 (Ser49-His63), β4-α5 loop (Lys93-Ser104), and the β6-α7 loop (Met123-Thr132) to have coincident bands in 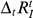 and 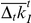. The sequentially collective bands highlighted in Figure 3(b,d) constitute a pathway of intra-protein communication, which is shown in Figure S5 and discussed in more detail in Text S1. The co-occurring bands of these loops in Ca^2+^-bound subtilisin, Figure 3(a,c), are less prominent and have different patterns, showing that Ca^2+^-mediated interactions alter the choreography of protein dynamics.

Along a similar line, as the β8–β9 loop (Gly153-Asp171) mechanically couples with the β6-α7 loop (Met123-Thr132) (Figure 1(d)), and the signal of Ca^2+^ binding propagates there accordingly. A clear difference between the fluctuograms of Ca^2+^-bound and apo subtilisin is that apo subtilisin has less prominent bands in β6-α7 and β8–β9 loops, opposite to the responses in β1–β2, β2-α3, and β4-α5 loops, Figure 3. Opposite responses of different loops to Ca^2+^-mediated interactions is reminiscent of the compensatory balance in mechanical coupling variation shown in Figure S2. The β8–β9 loop contains residues of the weaker Ca^2+^ binding site of subtilisin [44] and is 32 Å away from the strong Ca^2+^ binding site; fluctuogram analysis shows that through mechanical coupling network, signal at the Ca^2+^ binding site affects distal sites.

Other significant differences in the fluctuograms are that apo subtilisin has more pronounced 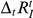 and 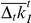 bands in the β10–β11 loop (Phe188-Ala193), the β12–β13 turn (Thr207-Tyr213), the α14–α15 loop (Lys236-Ala242), and the Phe260 turn (Gly257-Gly263), see highlights in Figure 3(b,d). These sites are also consistent with the results of 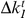
's shown in Figure 2(a).

Together, the fluctuograms calculated from all-atom MD simulations show that intra-protein communication can proceed through the mechanical coupling network in protein structure without a drastic conformational change [11], [45]. The results discussed above establish (a) Ca^2+^ binding induces significant changes in the mechanical coupling network of subtilisin despite a small difference in the overall structure, (b) residues with large mechanical coupling variation due to Ca^2+^ binding correlate with the gain-of-function mutation sites selected via directed evolution, (c) conformational changes and mechanical coupling variation are temporally and spatially intermittent, (d) large variations in the mechanical coupling network often occur at the connecting regions of secondary structures, and (e) the fluctuograms can be used to capture the pathways of intra-protein communication. To further strengthen (e), the sequentially collective conformational changes associated with the co-occurring bands highlighted in Figure 3 are discussed in Figure S4, Figure S5, and Text S1.

### Correlate fluctuograms with co-evolution

The fluctuograms of Ca^2+^-bound and apo subtilisin illustrate the mechanism of intra-protein communication and show that residues surviving from random mutagenesis and screening tend to have large mechanical coupling variation due to molecular signals. In theory, if the mechanical coupling network in protein structure was optimized by evolution to facilitate intra-protein communication, residue pairs with functionally important mechanical coupling would tend to correlate during evolution. To test this hypothesis, we select residue pairs with distinct patterns of mechanical coupling from the fluctuograms and compare the results with those of statistical coupling analysis (SCA). After collecting a pool of sequences with high similarity and constructing a multiple sequence alignment, the SCA method developed by Ranganathan and coworkers [37], [38] is used to identify residues with high sequence correlation.

**Figure 4:**
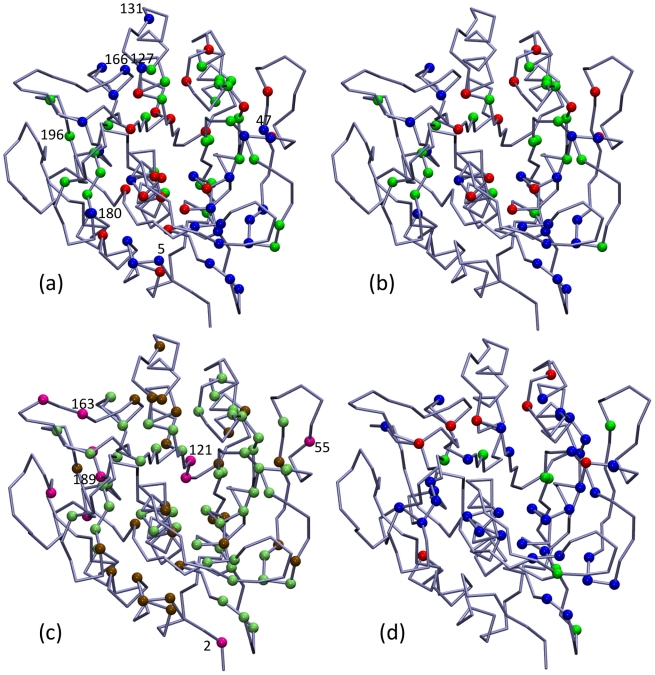
Sequence correlation in subtilisin. (a) The residues of subtilisin exhibit high correlation in our multiple sequence alignment determined by a statistical coupling analysis (SCA). Residues with high correlation in sequence variation are divided into three sectors, blue, red, and green according to the eigenvectors of the correlation matrix of sequence conservation [38]. Several residues that are not covered by the selection from the Ca^2+^-bound fluctuogram are highlighted. (b) The residues that satisfy either of the three criteria discussed in the text from the Ca^2+^-bound fluctuogram and cover the co-evolved residues shown in (a); color codes are the same as in (a). The parameters of the selection criteria are: 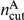 = 10, 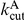 = 2.5, 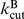 = 11, 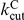 = 8.0, and 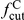 = 0.8. (c) The residues selected from the Ca^2+^-bound fluctuogram based on the parameters listed in (b). Lime: residues that cover the co-evolved residues from SCA. Brown: the co-evolved residues from SCA that are not covered by the residues selected from the Ca^2+^-bound fluctuogram. Pink: residues selected from the Ca^2+^-bound fluctuogram but do not cover any of the co-evolved residues. (d) The residues selected from the Ca^2+^-bound fluctuogram based on the parameters listed in (b). Blue: residues selected by Criterion-A. Red: residues selected from Criterion-B. Green: residues selected from Criterion-C. See text for the definitions of each criterion.

Using subtilisin Carlsberg as the query sequence, we collected 465 sequences for SCA (see methods for details), and the pattern of sequence conservation is shown in Figure S6. The 2^nd^–4^th^ eigenvectors were used to screen the correlation matrix for statistically significant correlation according to random matrix theory [38], [46]. The 274 residues of subtilisin expanded by the 2^nd^ and 3^rd^ eigenvectors are shown in Figure S7; on this map, a cutoff value of 0.07 for the distance to origin is used to select 80 residues (∼30% of the total amino acids) that exhibit high correlation in sequence variation [38]. The cleaned correlated matrix is shown in Figure S8. The 80 amino acids can be roughly divided into three sectors according to their values on the 2^nd^ and 3^rd^ eigenvectors, and their locations in subtilisin are shown in Figure 4(a). Spatial localization of sectors is rather clear but close separation of residues in different sectors is also observed. The pattern of sectors is consistent with several features of the long-range coupling and complex folding pathways of subtilisin [6], [24], [47]. For example, the blue sector contains residues in the Ca^2+^-binding loop (Val71-Leu83) and the weaker Ca^2+^ binding site, and analyzing the fluctuogram shows that the two Ca^2+^-binding sites are linked through the mechanical coupling network. Many red sector residues are localized in the central α3 and α14 of subtilisin (Figure 1(a)). The green sector contains residues in β1 (Val26-Leu31), the β1–β2 loop (Asp32-Asp41), and the β4-α5 loop (Lys93-Ser104) that mechanically couple with Asp32. At the junction of Asp32, the fluctuograms of apo and Ca^2+^-bound simulations show significant differences in Figure 3.

In recent years, significant progress has been made in connecting the network of protein structure to allosteric coupling [13], [14], [15], [16], [17], [18], [48], [49], [50], [51], [52]. Many of these studies employ ENM using contact-based determination of connectivity and heuristics-based assignment of force constants (homogeneous or via an assumed functional form) [13], [14], [15], [16], [17], [18]. Despite the simplicity, impressive success has been achieved in identifying important residues for allosteric coupling, which are often robust to sequence variation [53]. A key observation is that amino acids with many close contacts with others often have significant impact on allosteric coupling. Such residues are also considered as hubs that cause the structural network of protein conformation to have small-world characters [48], [50], [51], [52]. To select residue pairs from fluctuograms, we also apply this result developed in previous works.

The fluctuogram approach proposed in this work bridges atomic and CG models of protein allostery by computing the force constants in C_α_-SC-ENM from the structures sampled in all-atom MD simulations. An important result is that mechanical coupling between residues varies significantly, highlighting the anharmonicity and nonlinearity of protein dynamics. Therefore, both the strength and variation of mechanical coupling will be used to select residue pairs. From the 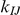
's calculated from sequential time windows, the average, 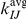, standard deviation, 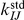, and maximum observed value, 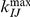, are computed to devise selection criteria.

In Criterion-A, we consider residue pars with 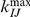 larger than a cut-off value, 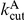. A value of 2.5 kcal/mol/Å^2^ was used earlier to assign whether the mechanical coupling between the *IJ* pair is significant. For residue *I*, the total number of coupled residues with 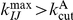 is denoted as 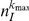. If 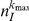 is larger than a number cut-off, 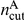, then residue *I* is selected as a residue important for intra-protein communication:
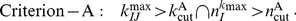
(2)The total number of such residues is denoted as 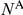. For each of the 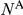 residues, if it captures any highly correlated residues observed in SCA to within ±1 in residue number, a hit is counted. The hit rate, 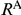, is calculated as the total number of hits, 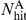, divided by 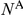, 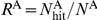. For each of the residues identified by SCA, we also check if it is covered by any of the 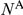 residues predicted by the fluctuogram. The total number of covered residues is 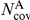, and the coverage is defined as 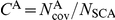. 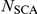 is the number of highly correlated residues identified in SCA.

**Figure 5:**
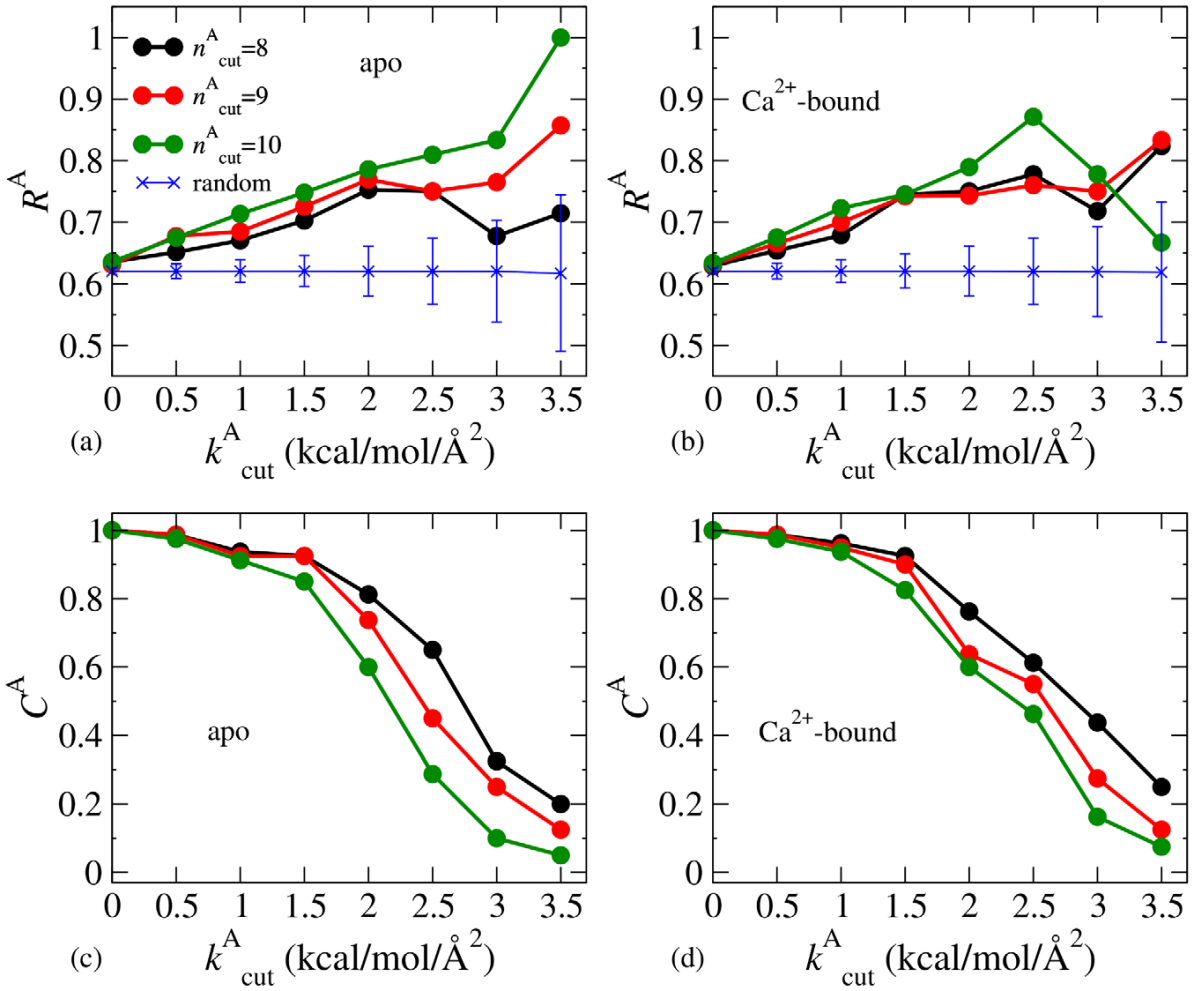
Correlating the fluctuograms of subtilisin with co-evolution. The calculated hit rates (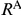
's) and coverages (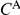
's) by using Criterion-A, (eq.(2)). (a) 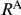
's from the apo fluctuogram. (b) 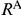
's from the Ca^2+^-bound fluctuogram. Hit rates achieved by randomly picking the same numbers as the selected residues based on 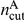 = 8 are shown for comparison. The profiles correspond to other 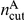 values are quantitatively close. (c) 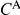
's from the apo fluctuogram. (d) 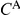
's from the Ca^2+^-bound fluctuogram.

The hit rates calculated from the fluctuograms of apo and Ca^2+^-bound subtilisin at different values of 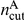 are shown in Figure 5(a) and Figure 5(b), respectively. At a given value of 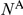, the hit rate achieved by randomly picking residues is also calculated for comparison (10,000 rounds; results of 1,000 rounds are quantitatively similar). In Figure 5(a,b), the hit rates of random picking correspond to the 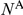 values of 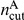 = 8; the profiles of other 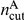 values are quantitatively similar. When 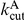 is small, the hit rates calculated from fluctuograms are close to the values of random picking. Since there are 80 highly correlated residues observed in SCA and a ±1 criterion is used for counting a hit, the baseline hit rate via random picking is 0.62. As shown in Figure 5(a,b), increasing 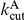 significantly improves the hit rates achieved by apo and Ca^2+^-bound fluctuograms, which are progressively higher than the values of random picking by more than one standard deviation. As 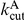 increases, 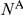 and the coverage decrease due to the more stringent selection. The coverages achieved by apo and Ca^2+^-bound fluctuograms are shown in Figure 5(c) and Figure 5(d), respectively. At small 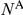 values, the standard deviation of the hit rates of random picking also becomes higher.

Figure 5(a,b) illustrate the correlation between mechanical coupling and co-evolution. The increasing hit rates with 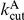 plateau around the value of 2.5 kcal/mol/Å^2^. This result is consistent with the physics-based selection of the value of 2.5 for assigning significant mechanical coupling. Overall, the hit rate is also an increasing function of 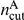, except for the special cases at small 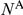 values. This trend is in line with the analyses of protein structure using network theory that residues with more neighbors tend to play important roles in allosteric coupling [48], [50], [51], [52]. In balancing hit rate and coverage, using 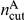 = 10 and 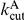 = 2.5 kcal/mol/Å^2^ for Criterion A gives 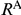 = 0.81 and 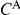 = 0.29 from the apo fluctuogram and 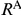 = 0.87 and 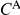 = 0.46 from the Ca^2+^-bound fluctuogram.

**Figure 6 pcbi-1002023-g006:**
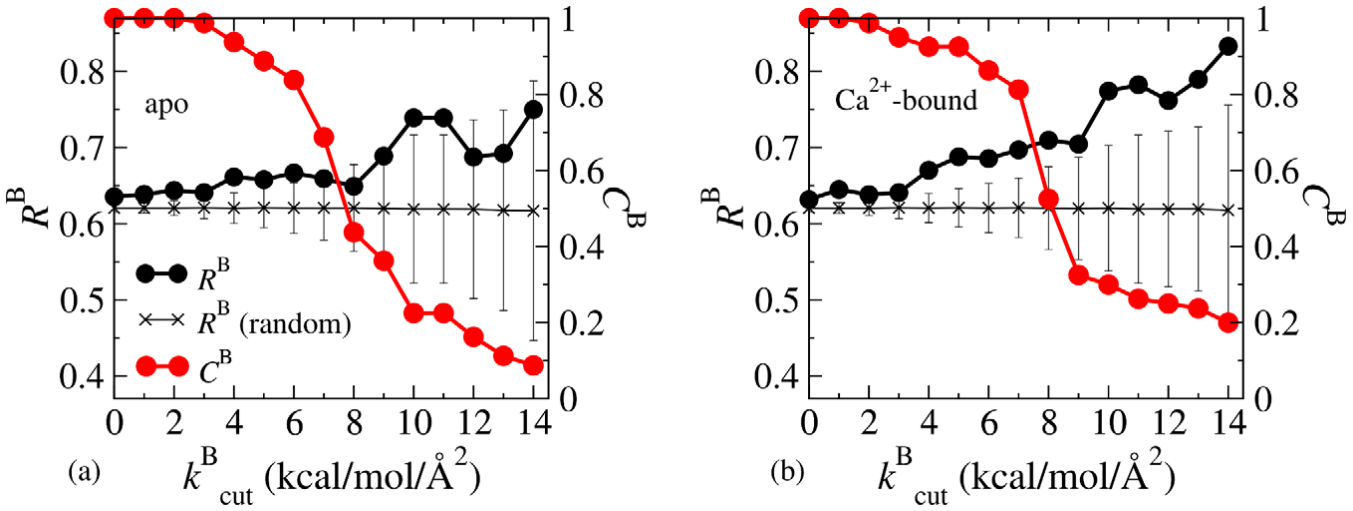
The calculated hit rates (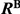
's) and coverages (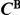
's) by using Criterion-B, (eq.(3)). (a) 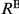
's and 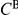
's from the apo fluctuogram. Hit rates achieved by randomly picking the same numbers as the selected residues are shown for comparison. (b) 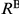
's and 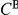
's from the Ca^2+^-bound fluctuogram.

In Criterion-B, we consider residue pairs with strong mechanical coupling. For any *IJ* pairs with 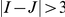, *I* and *J* are selected if:
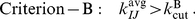
(3)The hit rates and coverages calculated from apo and Ca^2+^-bound fluctuograms are shown in Figure 6(a,b). Increasing 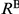 with 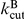 is also observed as in Criterion-A. The hit rates from the Ca^2+^-bound fluctuogram have steeper increase with 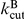 and exceed the values of random picking more than that from the apo fluctuogram. The coverage, 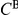, quickly decreases with 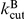, and is not as high as 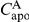, which screens 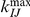 instead. For Criterion-B, we use 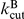 = 11 kcal/mol/Å^2^ (apo: 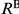 = 0.74 and 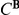 = 0.23; Ca^2+^-bound: 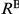 = 0.78 and 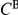 = 0.26).

**Figure 7 pcbi-1002023-g007:**
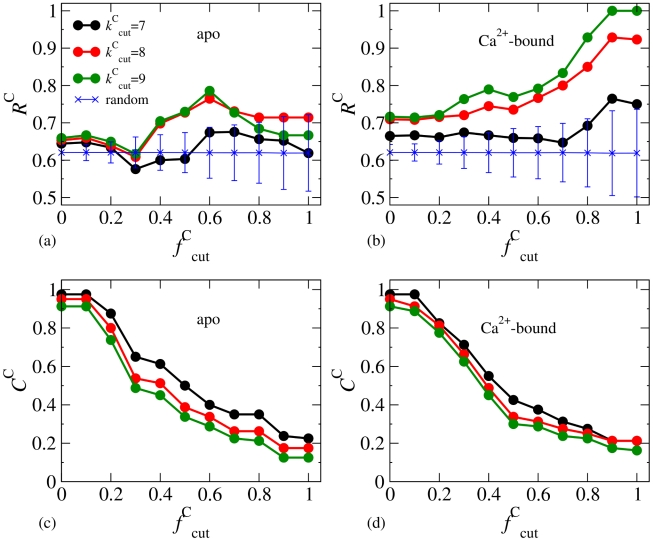
The calculated hit rates (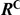
's) and coverages (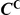
's) by using Criterion-C, (eq.(4)). (a) 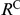
's from the apo fluctuogram. (b) 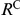
's from the Ca^2+^-bound fluctuogram. Hit rates achieved by randomly picking the same numbers as the selected residues based on 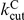 = 7 kcal/mol/Å^2^ are also shown for comparison. The profiles correspond to other 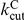 values are quantitatively close. (c) 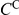
's from the apo fluctuogram. (d) 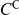
's from the Ca^2+^-bound fluctuogram.

In Criterion-C, we probe if the variation in 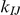 can capture the residues with high correlation in a multiple sequence alignment. In addition to limiting the magnitude of 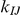, a cutoff for 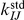 is also used:
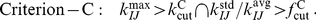
(4)Here, we employ 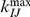 instead of 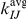 for the advantage of having higher coverage. The calculated 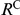
's and 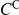
's are shown in Figure 7. From the apo fluctuogram, 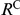 is not strictly increasing with 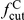, and the lead over random picking is only slightly higher or close to the average value plus standard deviation, Figure 7(a). From the Ca^2+^-bound fluctuogram, on the other hand, 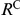 is clearly increasing with 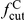, and the lead over random-picking values significantly exceeds the average plus a standard deviation, Figure 7(b). 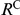 is also an increasing function with 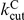 as expected from Criterion-A. For Criterion-C, we use 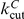 = 8 kcal/mol/Å^2^ and 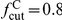 (apo: 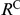 = 0.71 and 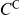 = 0.26; Ca^2+^-bound: 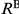 = 0.85 and 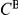 = 0.25).

As shown in Figure 2 and Figure 3 and discussed earlier, the fluctuogram of subtilisin depends on Ca^2+^ binding. As a result, different behaviors are observed in calculating hit rates from apo and Ca^2+^-bound fluctuograms. Since native subtilisin is functioning with Ca^2+^ and we screened for alignable sequences that contain the Ca^2+^-binding loop for SCA, the Ca^2+^-bound fluctuogram should better represent the required mechanical coupling network for the proper functioning of subtilisin. This theory is supported by the result that the Ca^2+^-bound fluctuogram has better predictive power in capturing the correlated residues from SCA. Using 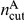 = 10, 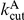 = 2.5, 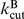 = 11, 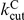 = 8.0, and 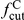 = 0.8 to select residues satisfying either criterion, the calculated hit rates and coverages are 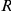 = 0.75/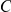 = 0.5 from the apo and 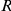 = 0.84/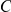 = 0.65 from the Ca^2+^-bound fluctuogram.

The correlated residues from SCA (Figure 4(a)) covered by the residue pairs with distinct behaviors of mechanical coupling in the Ca^2+^-bound fluctuogram are shown in Figure 4(b) for comparison. Several uncovered residues are highlighted in Figure 4(a) and many of them are in or near the pool of stabilization mutation sites shown in Figure 2(b). Therefore, comparing fluctuograms can provide additional information about co-evolution. The covered (green), missed (brown), and over-predicted (pink) residues based on the Ca^2+^-bound fluctuogram are contrasted in Figure 4(c), and several over-predicted residues are highlighted. Some of these residues are in or near the pool of the stabilization mutation sites shown in Figure 4(b) but are not selected in SCA. This result is consistent with many observations that thermodynamic coupling is not limited to co-evolved residues [54], [55], [56].

The increasing hit rates with the magnitude and variation of mechanical coupling link physics-based MD simulations with co-evolution. We devise different criteria to probe the properties of the mechanical coupling network in protein structure and to select residues to cover the correlated residues from SCA. Based on the Ca^2+^-bound fluctuogram, the covered SCA residues by using Criterion-A, Criterion-B, and Criterion-C together are colored differently in Figure 4(c) to illustrate that in the pool of residues with high sequence correlation, alternative behaviors of mechanical coupling are found.

**Figure 8:**
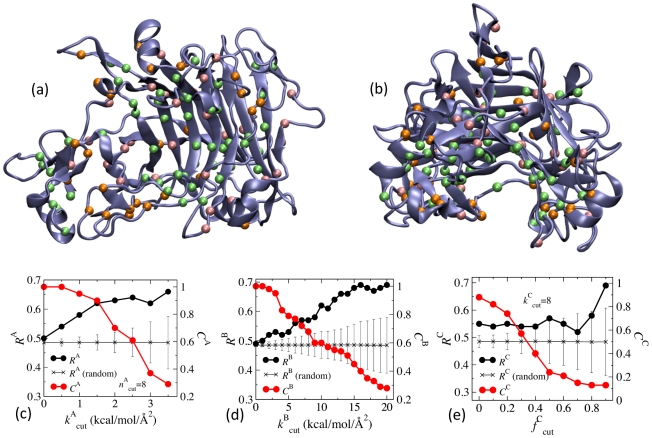
Correlating the fluctuograms of EG1 with co-evolution. (a) The highly correlated residues observed in a multiple sequence alignment and SCA using EG1 as the query sequence and the residues selected from the fluctuogram satisfying either of the three criteria with the following parameters: 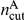 = 8, 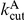 = 2.5, 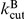 = 17, 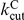 = 8.0, and 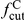 = 0.8. Lime: residues that cover the co-evolved residues from SCA. Brown: co-evolved residues from SCA that are not covered by the residues selected from the fluctuogram. Pink: residues selected from the fluctuogram but do not cover any co-evolved residue. (b) the same as (a) but view from a different angle. The calculated hit rates and coverages from the fluctuogram of EG1 by using (c) Criterion-A, (eq.(2)), (d) Criterion-B, (eq.(3)), and (e) Criterion-C, (eq.(4)).

As an independent test of the correlation between mechanical coupling and co-evolution, we analyze the fluctuograms of a different enzyme using the same criteria, in particular, the family 7 endoglucanase of the *Trichoderma reesei* fungus, EG1 [57]. The 371-residue EG1 hydrolyzes the β-1,4-glycosidic bonds in cellulose for nutritional utilization. To work against a glucose chain, EG1 has a tunnel-shape active site, Figure 8(a,b). The segments around the active site contain multiple secondary structures and connecting loop and are responsible for binding the glucose chain from the surface of cellulose. Therefore, the mechanical coupling network in EG1 needs to carry out non-catalytic activities, and correlating co-evolved residues via fluctuograms can reveal the functional relevance of the mechanical coupling network in EG1.

Using EG1 as the query sequence, we collected 318 sequences for SCA, and 82 residues with high correlation in sequence variation are identified and shown in Figure 8(a,b), see Methods for the details of methodology. The all-atom MD simulation of EG1 in explicit water at 300 K and 1 atm started with the X-ray structure, PDB ID 1EG1 [57], with the protocol described in Methods. The system contains 62,610 atoms, with 5256 protein atoms, 69 counter ions, and 19095 water molecules. A total of 80 ns trajectory was collected for calculating fluctuogram with 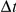 = 4 ns.

The calculated hit rates and coverages using Criterion-A, Criterion-B, and Criterion-C are shown in Figure 8(c), Figure 8(d), and Figure 8(e), respectively. The hit rates achieved by random picking are also shown for comparison. The increasing hit rates with 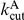 and 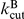 are clear in Figure 8(c,d), and the hit rates calculated from the fluctuogram exceed the mean values plus standard deviation of random picking to a large extent. In Criterion-C, the increase of hit rate with 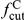 starts at larger values (Figure 8(e)). The hit rate calculated from the fluctuogram is higher than the mean values of random picking but not as much as in Criterion-A and Criterion-B. Similar behavior is also observed in calculating hit rates from the apo fluctuogram of subtilisin (Figure 7(a)). Currently, we are investigating the effects substrate binding on the mechanical coupling network in EG1. Using 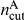 = 8, 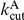 = 3.5, 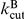 = 17, 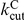 = 8.0, and 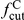 = 0.8, the covered, missed, and over-predicted residues compared to the co-evolved ones are shown in Figure 8(a,b).

The correlation between mechanical coupling and co-evolution in EG1 is clear in Figure 8. Therefore, in both subtilisin and EG1, the results of analyzing fluctuograms illustrate that the mechanical coupling networks calculated from atomic details can be used to correlate with co-evolution. Several noticeable differences between EG1 and subtilisin, though, can be found. First, residues in EG1 do not have as many neighbors with strong mechanical coupling, and a lower number 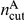 is thus used for Criterion-A. This result is consistent with the more globular shape of subtilisin. For Criterion-B, which screens for residue pairs with strong mechanical coupling on average, hit rates plateau at a larger 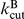 value in EG1 (17 kcal/mol/Å^2^) than that in subtilisin (11 kcal/mol/Å^2^). As EG1 is required to bind a polymer substrate already interacting with other molecules on the solid surface, strong mechanical strength in protein structure is likely needed for carrying out the required non-catalytic actives.

## Discussion

The mechanism of allosteric coupling and intra-protein communication is key to understand the structure-property relationship of protein function. An emergent picture is that induced-fit and population shift theories provide complementary pictures and do not exclude each other [11], [12], [13], [17], [58]. The interaction energetics between amino acids that cause induced-fit and the distribution of protein structures are the two sides of the same coin, and inverse algorithms such fluctuation matching [28] or the iterative Yvon-Born-Green [22] methods could be used to establish the connection. In this work, the fluctuation matching method is used to convert the configurations sampled in MD simulation into the bond lengths and force constants in a C_α_-SC-ENM to represent the mechanical coupling network in protein structure.

An important concern is the functional roles of the anharmonicity and nonlinearity in protein dynamics, especially in allosteric coupling without a drastic structural change. The population of similar but distinct protein structures may still shift due to molecular signals [11], [58] and harmonic models are not suitable for describing the concomitant reorganization of the mechanical coupling network. For subtilisin Carlsberg, the Ca^2+^-binding loop is distal to substrate-binding and active sites, yet the serine protease function depends on Ca^2+^ binding. Furthermore, there is no direct evidence that subtilisin forms alternative structures. Therefore, the intra-protein communication in subtilisin is likely related to the anharmonicity and nonlinearity of protein dynamics.

To test this hypothesis, we transform the sequential segments of an atomic MD trajectory into separate elastic network models. The anharmonicity and nonlinearity are thus effectively represented as the temporal and spatial variation of the mechanical coupling network. In analogy to the spectrogram of sound waves, the periodic transformations of structural fluctuations into ENMs are termed the “fluctuogram\, which records the choreography of protein dynamics. The fluctuograms of Ca^2+^-bound and apo subtilisin illustrate that local conformational changes and mechanical coupling variation are spatially and temporally intermittent: large changes at one location do not last long and different segments alternatively have prominent events between time windows (Figure 3). The fluctuograms also revealed the pathways of intra-protein communication. Ca^2+^-bound and apo subtilisin have distinct fluctuograms, illustrating that although a drastic structural change did not occur, Ca^2+^-mediated interactions caused significant effects at distal sites through the mechanical coupling network.

The Ca^2+^-dependent fluctuograms of subtilisin are in line with several experimental observations. In enhancing subtilisin stability by directed evolution and site-directed mutagenesis, it was found that certain mutations that stabilize apo subtilisin would destabilize the protein in the presence of Ca^2+^ [6]. Therefore, the mechanism of thermal inactivation depends on Ca^2+^ binding, implying that the mechanical coupling network in subtilisin is Ca^2+^ dependent. Indeed, our simulations show that apo and Ca^2+^-bound subtilisin have different fluctuograms. A mutation site of this type is Phe50 in the β2-α3 loop that shows different behaviors in Figure 3.

For modulating the functional properties of subtilisin via protein engineering, the strong Ca^2+^-dependence of stability and folding suggests that the residues with large mechanical coupling variation due to Ca^2+^-binding (Figure 2(a,b)) could be potential hot spots. Since the thermodynamic interactions associated with these residues are more susceptible, mutation of these residues should achieve the goal of altering protein stability. As mechanical coupling also affects conformational flexibility and dynamics, modulating the mechanical coupling network is also expected to change other functional properties such as substrate binding and activity. Many mutation sites that enhance the stability of subtilisin and its activity in an non-aqueous environment had been selected via random mutation and screening [31], [32], [33], [34], [35], [36], Figure 2(c), and are employed for testing the proposed connection between the mechanical coupling network and protein engineering of subtilisin. In Figure 2(a,b), we show that the residues calculated to have large mechanical coupling variation correlate with the reported mutation sites that had been shown to increase the stability and activity of subtilisin. As presented in Results, the agreement between the residues with large mechanical coupling variation and the reported gain-of-function mutation sites far exceeds that of randomly picking up the same number of residues. Therefore, the mechanical coupling networks calculated from atomic details can be used to correlate with functionally important mutation sites and a potential usage of fluctuograms is to identify the susceptible spots in a mechanical coupling network for protein engineering.

The fluctuogram analysis illustrates that the mechanical coupling network in protein structure is tightly coupled to functional properties such as stability and intra-protein communication. If the mechanical coupling network specified by sequence was optimized in addition to the structure, residue pairs with functionally important mechanical coupling would tend to correlate during evolution. To test this hypothesis, we devise criteria to select residue pairs from the fluctuograms and compare with those from a SCA on the results of a multiple sequence alignment [38].

Since the fluctuograms are calculated from atomic MD simulations, sequence specific properties of the mechanical coupling network are captured. Furthermore, the calculation does not require the knowledge of specific protein motions [14], [53]. We show in Figure 5–8 that the residues calculated to have distinctive behaviors of mechanical coupling can capture to a large extent the residues observed to have high correlation in a multiple sequence alignment. The results also indicate that the predictive power in capturing residues with high sequence correlation depends on the fluctuogram used for calculations. For subtilisin, the Ca^2+^-bound simulation is expected to better capture the functionally important mechanical coupling, since native subtilisin requires Ca^2+^ to work and globally alignable sequences with the presence of the Ca^2+^-binding loop are used for SCA. Indeed, the Ca^2+^-bound fluctuogram gives higher hit rates and coverages than the apo fluctuogram as shown in Figure 5–7. In addition to the magnitude of force constants, the variation of coupling strength is also found to be an indicative property for the sequence correlation observed in SCA. The robustness of using fluctuogram to capture residues with high sequence correlation is further tested with a different enzyme, EG1, and the results also show that the residues selected from the proposed criteria capture to a large extent the highly correlated residues observed in SCA (Figure 8(c–e)). Overall, our results illustrate that the mechanical coupling networks calculated from atomic details can be used to correlate with functionally important mutation sites and co-evolution.

The design of MD simulations with relevant scenarios and the criteria for selecting residues are the two core elements in using fluctuograms to study protein function and dynamics. We demonstrate that calculating fluctuograms as a function of molecular signal such as Ca^2+^ binding and comparing the resulting differences is a useful strategy to map out the residues that are important to the specific property conveyed by the probed signal. Therefore, calculating fluctuograms during the relevant rare events of protein function, such as enzymatic reaction, substrate binding, local unfolding [59], protein-protein association, and conformational changes in allostery is likely a useful strategy to establish the connection between a specific protein function and the fluctuograms of protein dynamics. This approach can be pursued by using multiscale computational methods such as reaction path optimization and free-energy simulations [60], [61] and is currently being explored in our laboratory.

Although the proposed procedure for calculating fluctuograms is general and can be applied to any set of MD trajectories, identifying residues important for function relies on the design of selection criteria. Several criteria based on the statistics of force constants are proposed heuristically and their abilities to correlate with co-evolution vary from one protein to another as illustrated by comparing the results of subtilisin and EG1. Such protein dependence is not unexpected given the complexity and specificity of protein sequence, structure, and function. In modeling complex allosteric protein systems that spans a diverse range, we envision that the success of applying the fluctuogram approach will not only depend on the design of relevant MD simulations but also on the criteria used for selecting function-related residues. Therefore, we are also developing systematic ways to partition and categorize the different behaviors of mechanical coupling in fluctuograms to better map out their connection with specific properties that are relevant to function. Direct comparison of the predicted residues with experimental measurements that provide amino-acid level information, such as by NMR methods, would be also valuable in validating and improving the selection criteria of fluctuogram analysis.

## Methods

### All-atom molecular dynamics simulations

We obtained the details of subtilisin by MD simulations using the CHARMM22 all-atom force field and the TIP3P water model [62], [63]. The particle mesh Ewald method [64] was used for calculating long-range electrostatics. For short-range non-bound interactions, a cutoff of 14 Å was used with a switch function turned on at 12 Å. Starting from an X-ray structure (PDB ID 1OYV) [5], a 100 ns trajectory was collected at 300 K and 1 atm after minimization (100,000 steps), heating (+4 K/ps over 100 ps via velocity reassignment), and equilibration (4 ns) steps. During minimization, heating, and the first 3 ns of equilibration, C_α_ atoms were restrained to their positions in the X-ray structure via harmonic potentials with a force constant of 1 kcal/mol/Å^2^. No restraint potentials or external forces were applied in the last ns of equilibration and production runs. Langevin dynamics with a damping coefficient of 0.5 ps^−1^ were used to maintain system temperature at 300 K [65], and the Langevin piston method was used to maintain pressure at 1 atm [66]. A time step of 2 fs was used to propagate dynamics simulations during which all covalent bonds associated hydrogen atoms were constrained at their equilibrium values defined in the CHARMM parameter. A Cl^−^ ion was added to neutralize the subtilisin system in the Ca^2+^-bound simulation; in the apo simulation, a Na^+^ ion was added. Apo subtilisin is marginally stable [4], [5], [6], [7]. A total of 6,767 and 6,768 water molecules were used to solvate subtilisin in a truncated octahedral unit cell for Ca^2+^-bound and apo simulations, respectively. Periodic boundary conditions were applied. All-atom MD simulations were performed using the NAMD software [67]. Normal mode analysis and other analyses were performed using the CHARMM software [68]. Figures of protein structures were prepared via VMD [69].

### Compute the mechanical coupling network in subtilisin from all-atom MD

Without loss of generality, we choose to describe the mechanical coupling between amino acids via a commonly used coarse-grained (CG) elastic network model (ENM) [26], [27]. In most applications of ENM, a protein structure is used to define connectivity with a distance cut-off and a universal force constant is often assigned to ignore atomic details other than the native structure [26], [27]. Despite its simplicity, homogeneous ENM is robust in predicting collective conformational changes [14], [70], [71], [72], [73], [74], [75] and the profile of atomic mean square fluctuations when comparing with crystallographic B-factors [26], [27], [75]. More sophisticated schemes for determining force constants have been developed to improve the prediction of B-factors [76].

In our implementation, the sidechain and backbone contributions are treated separately by using two CG sites per amino acid. To determine the coordinates of CG sites from an atomic configuration, the C_α_ positions are used to define backbone sites and the centers of mass of sidechain atoms are used to define the sidechain sites; glycine has a single site. The mass of the backbone site is the total mass of all backbone atoms and the mass of the sidechain site is the total mass of all sidechain atoms. The resulting CG model is referred to as C_α_-SC-ENM (SC≡sidechain).

The force constant between two CG sites designates the strength of mechanical coupling. Each bond is treated separately and can have a different value. The potential energy function of the C_α_-SC-ENM is:
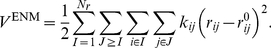
(5)In eq.(5), *I* and *J* are indices for residues; *N_r_* is the total number of residues in subtilisin. *i* and *j* are indices for CG sites; 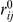 and 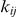 are the length and force constant of the elastic bond between sites *i* and *j*. The vibrational partition function corresponding to the potential energy function of eq.(5) can be computed via normal model analysis (NMA), and the predicted variance of each bond, 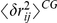, can be determined at a specified temperature [75], [77]. However, the statistics computed from a segment of an all-atom MD trajectory, 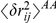, may be different. The fluctuation matching approach adjusts 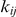
's iteratively to reduce the difference between 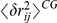 and 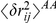:
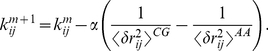
(6)In eq.(6), *m* is the step of fluctuation matching iteration, and *α* is a numerical constant. Each step requires a NMA on the C_α_-SC-ENM to update force constants. The fluctuations of each bond are approximated via Gaussian statistics and only non-negative force constants are used. Starting from an initial distribution of 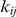 inversely proportional to 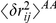, convergence (root-of-mean-square difference in force constants between steps <0.005 kcal/mol/Å^2^) is typically achieved within 200 steps.

In the example of subtilisin Carlsberg (Figure 1(a)), the 274-residue serine protease results in a 504-site C_α_-SC-ENM (incept in Figure 1(c)) with a specific site dedicated for Ca^2+^ (orange ball). All site pairs that have been within 10 Å during the course of 100 ns all-atom trajectory are included in the pool of elastic bonds for fluctuation matching. Since the force constants are adjusted according to eq.(6) to match the statistics of inter-site distances from all-atom MD, the results of fluctuation matching are not sensitive to the distance cutoff used for assigning initial connectivity. A cutoff of 10 Å provides sufficiently large basis for capturing inter-site mechanical coupling.

### Multiple sequence alignment and statistical coupling analysis

Subtilisin homologs were gathered from the NCBI's non-redundant database using GGSEARCH of the FASTA suite [78] as well as Pfam [79]. Sequences from the Pfam peptidase inhibitor I9 domain (PF05922) and the subtilase family domain (PF00082) were combined with the results from GGSEARCH. Since GGSEARCH returns only globally alignable sequences, both the mature protein sequence (274 aa) and the sequence including the N-terminal signal peptide and propeptide (379 aa) were used as queries. For both the GGSEARCH and Pfam sequences, an initial alignment was constructed with MAFFT [80], and then truncated to positions in the mature subtilisin Carlsberg structure (PDB 1OYV) [5]. Sequences were selected from these truncated alignments based on the number of alignable positions (no more than 100 gaps) and the presence of the Ca^2+^-binding loop (at most one gap at positions 75–79). After removing redundant sequences (≥95% similar) identified by BLASTClust [81], the sequences were re-aligned and the resulting alignment of 465 sequences was used for conducting the statistical coupling analysis. The statistical coupling matrix was created as described in Halabi et al. [38] and eigenvectors 2 and 3 of this matrix were used to assign three sectors. The cleaned SCA matrix (Figure S8) is used to visualize the sectors and was generated from eigenvectors 2 through 4. The endoglucanse SCA was conducted in a similar fashion. Our Mathematica code is based on the MATLAB code from [38] and is available upon request.

## Supporting Information

Text S1 Discussion of the sequentially collective conformational changes shown in Figure S4 and Figure S5.
(DOC)

Figure S1 The root-mean-square difference (RMSD) of the C_α_ atoms in Ca^2+^-bound (black) and apo (red) trajectories of subtilisin to the X-ray structure (PDB ID: 1OYV). The cross RMSD between the two simulations at each time frame is also shown in blue.
(EPS)

Figure S2The time evolution of the total force constant, *k_TOT_*, of Ca^2+^-bound and apo subtilisin. *k_TOT_* is the sum of all force constants between CG sites. The time window 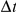 for calculating force constants is 4 ns.(TIF)

Figure S3Mechanical coupling variation in subtilisin due to Ca^2+^ binding. Variation in the force constant of each residue between neighboring time windows for (c) the Ca^2+^ simulation and (d) the apo simulation. The time window 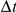 for calculating force constants is 4 ns.(PDF)

Figure S4 The time course of C_α_-C_α_ distances in Å between selected residue pairs for the Ca^2+^-bound (top) and apo (bottom) simulations. The trajectories of *d*(Val51-Leu95), *d*(Ser100-Gly127), and *d*(Gly127-Tyr166) of the Ca^2+^-bound simulation illustrate the sequentially collective conformational change corresponding to the highlighted band in fluctuogram shown in Figure 3(a,c). In the apo simulation, such conformational change was not observed.
(EPS)

Figure S5 The time course of C_α_-C_α_ distances in Å between selected residue pairs for the Ca^2+^-bound (top) and apo (bottom) simulations, (a) *d*(Asp41-Leu74), (b) *d*(Asp41-Val80), (c) *d*(Ala37-Asn43), (d) *d*(Ala37-Thr210), (e) *d*(Ile35-Als91), (f) *d*(Ile35-Asp59), (g) *d*(Ile35-Thr65), (h) *d*(Ile35-Asn57), (i) *d*(Gly99-Gly127, and (j) *d*(Val51-Asn96). The distance trajectories illustrate the sequentially collective conformational change in the apo simulation that occurred ∼50 ns. The corresponding bands in fluctuogram are highlighted in Figure 3(b,d). In the Ca^2+^-bound simulation, such conformational change was not observed.
(EPS)

Figure S6Positional conservation of the multiple sequence alignment, defined as the relative entropy between the observed amino acid frequencies *f* ^(*a*)^ in each column *i* and the background frequencies *q* ^(*a*)^ from all proteins: 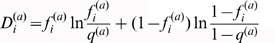. Following [33], a binary approximation was applied. Each position is represented as 1 if it contains the most prevalent amino acid in that column, or 0 otherwise. Columns are colored based on the clusters shown in Figure S7.(TIF)

Figure S7 Scatter plot of the 2^nd^ and 3^rd^ eigenvectors. A cutoff distance of 0.07 from the origin was used to select 80 residues that tend to co-evolve, which were divided into three clusters: blue, red, and green. Residues at a distance of 0.07–1.0 from the origin are colored with a lighter shade.
(TIF)

Figure S8 The statistical coupling matrix, calculated as described in [33]. Eigenvectors 2–4 were used for matrix cleaning and the matrix is truncated to the 80 positions appearing in the cluster analysis. Columns are grouped by cluster (in the order blue, red, and green). Within each cluster, positions are ordered by their distance from the origin along the 2^nd^ and 3^rd^ eigenvectors (Figure S7).
(TIF)

Video S1 (MPG)

Video S2 (MPG)

## References

[1] Koshland DE (1958). Application of a theory of enzyme specificity to protein synthesis.. Proc Natl Acad Sci USA.

[2] Monod J, Wyman J, Changeux JP (1965). An nature of allosteric transitions - A plausible model.. J Mol Biol.

[3] Yu EW, Koshland DE (2001). Propagating conformational changes over long (and short) distances in proteins.. Proc Natl Acad Sci USA.

[4] Kraut J (1977). Serine proteases - Structure and mechanism of catalysis.. Annu Rev Biochem.

[5] Barrette-Ng IH, Ng KKS, Cherney MM, Pearce G, Ryan CA (2003). Structural basis of inhibition revealed by a 1 ∶ 2 complex of the two-headed tomato inhibitor-II and subtilisin Carlsberg.. J Biol Chem.

[6] Bryan PN (2000). Protein engineering of subtilisin.. BBA-Protein Struct M.

[7] Wells JA, Estell DA (1988). Subtilisin - An enzyme designed to be engineered.. Trends Biochem Sci.

[8] Smock RG, Gierasch LM (2009). Sending signals dynamically.. Science.

[9] Ma B, Nussinov R (2009). Amplification of signaling via cellular allosteric relay and protein disorder.. Proc Natl Acad Sci USA.

[10] McNicholl ET, Das R, SilDas S, Taylor SS, Melacini G (2010). Communication between tandem camp binding domains in the regulatory subunit of protein kinase A-I alpha as revealed by domain-silencing mutations.. J Biol Chem.

[11] Whitley MJ, Lee AL (2009). Frameworks for understanding long-range intra-protein communication.. Curr Protein Pept Sci.

[12] Tsai C-J, del Sol A, Nussinov R (2009). Protein allostery, signal transmission and dynamics: a classification scheme of allosteric mechanisms.. Mol Biosyst.

[13] Cui Q, Karplus M (2008). Allostery and cooperativity revisited.. Protein Sci.

[14] Zheng WJ, Brooks BR, Thirumalai D (2006). Low-frequency normal modes that describe allosteric transitions in biological nanomachines are robust to sequence variations.. Proc Natl Acad Sci USA.

[15] Chennubhotla C, Bahar I (2006). Markov propagation of allosteric effects in biomolecular systems: application to GroEL-GroES.. Mol Syst Biol.

[16] Del Sol A, Tsai C-J, Ma B, Nussinov R (2009). The Origin of Allosteric Functional Modulation: Multiple Pre-existing Pathways.. Structure.

[17] Bahar I, Lezon TR, Yang L-W, Eyal E (2010). Global Dynamics of Proteins: Bridging Between Structure and Function.. Annu Rev Biophys.

[18] Sherwood P, Brooks BR, Sansom MS (2008). Multiscale methods for macromolecular simulations.. Curr Opin Struct Biol.

[19] Hilser VJ, Dowdy D, Oas TG, Freire E (1998). The structural distribution of cooperative interactions in proteins: Analysis of the native state ensemble.. Proc Natl Acad Sci USA.

[20] Hilser VJ, Garcia-Moreno B, Oas TG, Kapp G, Whitten ST (2006). A statistical thermodynamic model of the protein ensemble.. Chem Rev.

[21] Noid WG, Chu J-W, Ayton GS, Krishna V, Izvekov S (2008). The multiscale coarse-graining method. I. A rigorous bridge between atomistic and coarse-grained models.. J Chem Phys.

[22] Cho H, Chu J-W (2009). Inversion of Radial Distribution Functions to Pair Forces by Solving the Yvon-Born-Green Equation Iteratively.. J Chem Phys.

[23] Lee S, Jang DJ (2000). Cation-binding sites of subtilisin Carlsberg probed with Eu(III) luminescence.. Biophys J.

[24] Alexander PA, Ruan B, Bryan PN (2001). Cation-dependent stability of subtilisin.. Biochemistry.

[25] Gallagher T, Bryan PN, Gilliland GL (1993). Calcium-independent subtilisin by design.. Proteins.

[26] Tirion MM (1996). Large amplitude elastic motions in proteins from a single-parameter, atomic analysis.. Phys Rev Lett.

[27] Bahar I, Atilgan AR, Jernigan RL, Erman B (1997). Understanding the recognition of protein structural classes by amino acid composition.. Proteins.

[28] Chu J-W, Voth GA (2006). Coarse-grained modeling of the actin filament derived from atomistic-scale simulations.. Biophys J.

[29] Lyman E, Pfaendtner J, Voth GA (2008). Systematic Multiscale Parameterization of Heterogeneous Elastic Network Models of Proteins.. Biophys J.

[30] Johnson K (2003). Acoustic and Auditory Phonetics.

[31] Zhao H, Arnold F (1999). Directed evolution converts subtilisin E into a functional equivalent of thermitase.. Protein Eng.

[32] Strausberg S, Alexander P, Gallagher D, Gilliland G, Barnett B (1995). Directed evolution of a subtilisin with calcium-independent stability.. Biotechnology.

[33] Strausberg S, Ruan B, Fisher K, Alexander P, Bryan P (2005). Directed coevolution of stability and catalytic activity in calcium-free subtilisin.. Biochemistry.

[34] Rollence ML, Filpula D, Pantoliano MW, Bryan PN (1988). Engineering thermostability in subtilisin BPN' by in vitro mutagenesis.. CRC Crit Rev Biotech.

[35] Chen K, Arnold F (1991). Enzyme engineering for nonaqueous solvents - random mutagenesis to enhance activity of subtilisin-E in polar organic media.. Biotechnology.

[36] Chen K, Arnold F (1993). Tuning the activity of an enzyme for unusual environments - sequential random mutagenesis of subtilisin-E for catalysis in dimethylformamide.. Proc Natl Acad Sci USA.

[37] Suel GM, Lockless SW, Wall MA, Ranganathan R (2003). Evolutionarily conserved networks of residues mediate allosteric communication in proteins.. Nat Struct Biol.

[38] Halabi N, Rivoire O, Leibler S, Ranganathan R (2009). Protein sectors: Evolutionary units of three-dimensional structure.. Cell.

[39] Zhou HX (2004). Polymer models of protein stability, folding, and interactions.. Biochemistry.

[40] Tracewell CA, Arnold FH (2009). Directed enzyme evolution: climbing fitness peaks one amino acid at a time.. Curr Opin Chem Biol.

[41] Eppler RK, Komor RS, Huynh J, Dordick JS, Reimer JA (2006). Water dynamics and salt-activation of enzymes in organic media: Mechanistic implications revealed by NMR spectroscopy.. Proc Natl Acad Sci USA.

[42] Eppler RK, Hudson EP, Chase SD, Dordick JS, Reimer JA (2008). Biocatalyst activity in nonaqueous environments correlates with centisecond-range protein motions.. Proc Natl Acad Sci USA.

[43] Hudson EP, Eppler RK, Beaudoin JM, S. DJ, Reimer JA (2009). Active-Site Motions and Polarity Enhance Catalytic Turnover of Hydrated Subtilisin Dissolved in Organic Solvents.. J Am Chem Soc.

[44] Pantoliano MW, Whitlow M, Wood JF, Rollence ML, Finzel BC (1988). The engineering of binding-affinity at metal-ion binding-sites for the stabilization of proteins - Subtilisin as a test case.. Biochemistry.

[45] Tsai C-J, Del Sol A, Nussinov R (2008). Allostery: Absence of a change in shape does not imply that allostery is not at play.. J Mol Biol.

[46] Plerou V, Gopikrishnan P, Rosenow B, Amaral LAN, Guhr T (2002). Random matrix approach to cross correlations in financial data.. Phys Rev E.

[47] Fisher KE, Ruan B, Alexander PA, Wang L, Bryan PN (2007). Mechanism of the kinetically-controlled folding reaction of subtilisin.. Biochemistry.

[48] Daily MD, Upadhyaya TJ, Gray JJ (2008). Contact rearrangements form coupled networks from local motions in allosteric proteins.. Proteins.

[49] Vendruscolo M, Paci E, Dobson CM, Karplus M (2001). Three key residues form a critical contact network in a protein folding transition state.. Nature.

[50] Vendruscolo M, Dokholyan NV, Paci E, Karplus M (2002). Small-world view of the amino acids that play a key role in protein folding.. Phys Rev E.

[51] del Sol A, Fujihashi H, Amoros D, Nussinov R (2006). Residues crucial for maintaining short paths in network communication mediate signaling in proteins.. Mol Syst Biol.

[52] Ghosh A, Vishveshwara S (2007). A study of communication pathways in methionyl-tRNA synthetase by molecular dynamics simulations and structure network analysis.. Proc Natl Acad Sci USA.

[53] Zheng W, Brooks BR, Thirumalai D (2009). Allosteric transitions in biological nanomachines are described by robust normal modes of elastic networks.. Curr Protein Pept Sc.

[54] Fodor AA, Aldrich RW (2004). On evolutionary conservation of thermodynamic coupling in proteins.. J Biol Chem.

[55] Chi CN, Elfstrom L, Shi Y, Snall T, Engstrom A (2008). Reassessing a sparse energetic network within a single protein domain.. Proc Natl Acad Sci USA.

[56] Liu Z, Chen J, Thirumalai D (2009). On the accuracy of inferring energetic coupling between distant sites in protein families from evolutionary imprints: Illustrations using lattice model.. Proteins.

[57] Kleywegt G, Zou J, Divne C, Davies G, Sinning I (1997). The crystal structure of the catalytic core domain of endoglucanase I from Trichoderma reesei at 3.6 angstrom resolution, and a comparison with related enzymes.. J Mol Biol.

[58] Csermely P, Palotai R, Nussinov R (2010). Induced fit, conformational selection and independent dynamic segments: an extended view of binding events.. Trends Biochem Sci.

[59] Brokaw J, Chu J-W (2010). On the Roles of Substrate Binding and Hinge Unfolding in Conformational Changes of Adenylate Kinase.. Biophys J.

[60] Brokaw JB, Haas KR, Chu J-W (2009). Reaction Path Optimization with Holonomic Constraints and Kinetic-Energy Potentials.. J Chem Theory Comput.

[61] Haas RK, Chu J-W (2009). Decomposition of energy and free energy changes by following the flow of work along reaction path.. J Chem Phys.

[62] Mackerell AD (2004). Empirical force fields for biological macromolecules: Overview and issues.. J Comput Chem.

[63] Mackerell AD, Feig M, Brooks CL (2004). Extending the treatment of backbone energetics in protein force fields: limitations of gas-phase quantum mechanics in reproducing protein conformational distributions in molecular dynamics simulations.. J Comput Chem.

[64] Darden T, York D, Pederson L (1993). Particle mesh Ewald: an Nlog(N) method for Ewald sums in large systems.. J Chem Phys.

[65] Allen MP, Tildesley DJ (1987). Computer Simulation of Liquids.

[66] Feller SE, Zhang YH, Pastor RW, Brooks BR (1995). Constant-pressure molecular-dynamics simulation - The Langevin piston method.. J Chem Phys.

[67] Phillips JC, Braun R, Wang W, Gumbart J, Tajkhorshid E (2005). Scalable molecular dynamics with NAMD.. J Comput Chem.

[68] Brooks BR, Brooks CL, Mackerell AD, Nilsson L, Petrella RJ (2009). CHARMM: The Biomolecular Simulation Program.. J Comput Chem.

[69] Humphrey W, Dalke A, Schulten K (1996). VMD: Visual molecular dynamics.. J Mol Graphics.

[70] Lezon TR, Sali A, Bahar I (2009). Global Motions of the Nuclear Pore Complex: Insights from Elastic Network Models.. Plos Comput Biol.

[71] Ming D, Wall ME (2005). Allostery in a coarse-grained model of protein dynamics.. Phys Rev Lett.

[72] Maragakis P, Karplus M (2005). Large amplitude conformational change in proteins explored with a plastic network model: Adenylate kinase.. J Mol Biol.

[73] Chu J-W, Voth GA (2007). Coarse-grained free energy functions for studying protein conformational changes: A double-well network model.. Biophys J.

[74] Tama F, Valle M, Frank J, Brooks CL (2003). Dynamic reorganization of the functionally active ribosome explored by normal mode analysis and cryo-electron microscopy.. Proc Natl Acad Sci USA.

[75] Ma JP (2005). Usefulness and limitations of normal mode analysis in modeling dynamics of biomolecular complexes.. Structure.

[76] Yang L, Song G, Jernigan RL (2009). Protein elastic network models and the ranges of cooperativity.. Proc Natl Acad Sci USA.

[77] Brooks BR, Janezic D, Karplus M (1995). Harmonic-analysis of large systems .1. methodology.. J Comput Chem.

[78] Pearson WR, Lipman DJ (1988). Improved tools for biological sequence comparison.. Proc Natl Acad Sci USA.

[79] Finn RD, Mistry J, Tate J, Coggill P, Heger A (2010). The Pfam protein families database.. Nucleic Acids Res.

[80] Katoh K, Kuma K, Toh H, Miyata T (2005). MAFFT version 5: improvement in accuracy of multiple sequence alignment.. Nucleic Acids Res.

[81] Altschul SF, Madden TL, Schaffer AA, Zhang JH, Zhang Z (1997). Gapped BLAST and PSI-BLAST: a new generation of protein database search programs.. Nucleic Acids Res.

