# Supplementary material for: “Fluctuograms” Reveal the Intermittent Intra-Protein Communication in Subtilisin Carlsberg and Correlate Mechanical Coupling with Co-Evolution
Source: PLoS Comput Biol. 2011 Mar 24;7(3):e1002023. doi: 10.1371/journal.pcbi.1002023 (PMC3063751; doi:10.1371/journal.pcbi.1002023)
Supplement: Text S1 — Discussion of the sequentially collective conformational changes shown in Figure S4 and Figure S5. (DOC) [file pcbi.1002023.s001.doc]

*Compensatory balance in mechanical coupling variation*

Examining *k_TOT_*, the sum of all force constants between protein sites in C_α_-SC-ENM, indicates a compensatory balance in mechanical coupling variation. The time evolution of for Ca^2+^-bound and apo simulations are shown in Figure S2; both do not show systematic drift over 100 ns. The standard deviation is less than 0.4% of the average. The of apo subtilisin is only slightly smaller than that of Ca^2+^-bound subtilisin: the relative difference in average is only 0.5%. The force constants of individual amino acids, however, can differ by more than ±50 % between Ca^2+^-bound and apo subtilisin (Figure 2(a)). Therefore, Ca^2+^-mediated interactions cause significant variation in mechanical coupling network without a drastic change in . Adding the force constants between Ca^2+^ and protein sites ( only involves force constants between protein sites), though, make Ca^2+^-bound subtilisin more stable.

*Sequentially collective conformational changes for intra-protein communication*

The co-occurring and bands highlighted in Figure 3 can be associated with sequentially collective conformational changes and residue pairs with significant mechanically coupling are involved. The fluctuograms shown in Error: Reference source not found provide a systematic way to identify pathways of intra-protein communication.

Without loss of generality, we use C_α_-C_α_ distances between mechanically coupled residues to represent conformational changes. In the Ca^2+^-bound simulation, the conformational change associated with the highlighted region in Figure 3(a,c) is represented by the time evolution of *d*(Val51-Leu95), *d*(Ser100-Gly127), and *d*(Gly127-Tyr166) shown in Figure S4. An ~3 Å increase in *d*(Val51-Leu95), the C_α_-C_α_ distance between Val51 and Leu95, occurred around 45 ns, which lasted for ~7 ns and then returned back to around the original value. This event affected the mechanical coupling between the β4-α5 loop (Lys93-Ser104) and the β6-α7 loop (Met123-Thr132) on the other side. As a result, *d*(Ser100-Gly127) increased by ~4 Å from 55-70 ns; afterwards this distance has larger fluctuations. As the β6-α7 loop is mechanically coupled with the β8-β9 loop (Gly153- Asp171), *d*(Gly127-Tyr166) started to decrease at 55 ns with the increase in *d*(Ser100-Gly127). In the apo simulation, coupled changes in these distances were not observed as shown in Figure S4.

In the apo simulation, fluctuogram analysis shown in Figure 3(b,d) suggests a pathway of intra-protein communication radiating from the β1-β2 loop, and several C_α_-C_α_ distances are selected to describe this conformational change induced by Ca^2+^ binding. The time evolutions of *d*(Asp41-Leu74) in Ca^2+^-bound and apo simulations are shown in Figure S5(a). Leu74 is in the Ca^2+^-binding loop and Asp41 coordinates Ca^2+^ if present. Longer *d*(Asp41-Leu74) in apo subtilisin is clear. On the other side of Asp41, apo subtilisin has shorter *d*(Asp41-Asp80), Figure S5(b), indicating that the relative orientation of the Ca^2+^-binding loop to Asp41 is altered due to Ca^2+^ binding. Fluctuations of both distances in the apo simulation are also larger.

Asp41 is located at the junction between β1-β2 loop (Asp32-Asp41) and β2 (Leu42-Ala48), the absence of Ca^2+^-mediated interactions affected the packing of the β1-β2 loop against β2: the distance separation between them increased in the apo simulation as seen in the time evolution of *d*(Ala37-Asn43), Figure S5(c). Starting ~50 ns in the apo simulation, *d*(Ala37-Asn43) increased 4 Å in 3 ns; in the Ca^2+^-bound simulation, this distance remained constant instead. Another signature of the β1-β2 loop conformational change is the decreasing *d*(Ala37-Thr210) after 50 ns, Figure S5(d). The evolution of these two distances indicate that the β1-β2 loop moved away from β2 and closer to the β12-β13 turn (Tyr208-Thr212) as a result of Ca^2+^ binding.

Further away from the Ca^2+^-binding loop, the packing around Ile35 in the β1-β2 loop also changed due to Ca^2+^ binding: in the apo simulation, *d*(Ile35-Ala91) increased with that of *d*(Ala37-Asn43) starting ~50 ns (Figure S5(c,e)). Asn43 is in β2 and Ala91 is in β4. The β1-β2 loop thus moved away from both β2 and β4 as a result of Ca^2+^ binding. On the other side, Ile35 is mechanically coupled with the β2-α3 loop (Ser49-His63) and *d*(Ile35-Asp59) decreased as the β1-β2 loop moved away from β2 and β4, Figure S5(f).

In the apo simulation, the β1-β2 loop conformational change due to Ca^2+^-binding led to the moving away from β2 and β4 as shown in Figure S5(c-f). Concomitant changes in the packing of the β1-β2 loop with respect to α3 in the left-handed β2-α3-β4 motif further illustrate the connection between mechanical coupling and conformational changes. When the β1-β2 loop moved away from β2 and β4 in the apo simulation, *d*(Ile35-Thr65) started to increase at 50 ns as shown in Figure S5(g); Thr65 is in the N-terminal of α3 (Gly64-Thr70). With a strong mechanical coupling between Ile35 and Thr65, the Thr65 portion of α3 quickly moved with the β1-β2 loop such that *d*(Ile35-Thr65) decreased back to a value that is only lightly above the original value at 74 ns, Figure S5(g). On the other side of Ile35 with respect to Thr65 is Asn57 in the β2-α3 loop (Ser49-His63), and the slight increase in *d*(Ile35-Thr65) led to a slight decrease in *d*(Ile35-Asn57) starting 74 ns, Figure S5(h). Therefore, the packing of the β1-β2 loop against the β2-α3 loop also varied after the β1-β2 loop conformational change.

The β4-α5 loop (Lys93-Ser104) is mechanically coupled to β1-β2 (Asp32-Asp41) as well as β2-α3 (Ser49-His63) loops at the farther end of the Ca^2+^-binding site (Figure S5(d)), and the aforementioned conformational changes induced by Ca^2+^-binding eventually affected the β4-α5 loop. As *d*(Ile35-Thr65) and *d*(Ile35-Asn57) approached their plateau values ~74 ns in the apo simulation (Figure S5(g,h)), the β4-α5 loop began to shift toward the β6-α7 loop (Met123-Thr132) as evidenced by a 5 Å decrease in *d*(Gly99-Gly127) (Figure S5(i)) and an increase in *d*(Val51-Asn96) on the other side, (Figure S5(j)).

The sequentially collective (stepwise) conformational change shown in Figure S5 corresponds to an intra-protein communication pathway through mechanically coupled residue pairs. This Asp41-Asn43-Ala37-Ile35-Leu95-Gly99-Gly127 path is shown pictorially in Figure 1(a) via orange arrows. The movies made from the C_α_-SC-ENMs of Ca^2+^-bound and apo fluctuograms are provided as VideoS1 and VideoS2, respectively.

The β1-β2 loop conformational change discussed above due to Ca^2+^ binding did not occur until 50 ns in the apo simulation (Figure S5(c,d)), and the corresponding and bands appeared ~54 ns in Figure 3(b,d). Beforehand, local conformational changes and mechanical coupling variation in the loops discussed earlier (Figure 3(b,d)) occurred as preorganization for the conformational change, with patterns distinct from those in the Ca^2+^-bound simulation, Figure 3(a,c). In Figure 3, gaps separating the events of and bands are frequently observed. The intermittent patterns of local conformational change and mechanical coupling variation suggest that preorganization and reorganization are constantly ongoing during protein dynamics.
