# Supplementary figures and images for: “Fluctuograms” Reveal the Intermittent Intra-Protein Communication in Subtilisin Carlsberg and Correlate Mechanical Coupling with Co-Evolution
Source: PLoS Comput Biol. 2011 Mar 24;7(3):e1002023. doi: 10.1371/journal.pcbi.1002023 (PMC3063751; doi:10.1371/journal.pcbi.1002023)

(a)  $\text{Ca}^{2+}$ -bound

$$\Delta_t k_I^t = k_I^t - k_I^{t-1/2\Delta t}$$

apo (b)

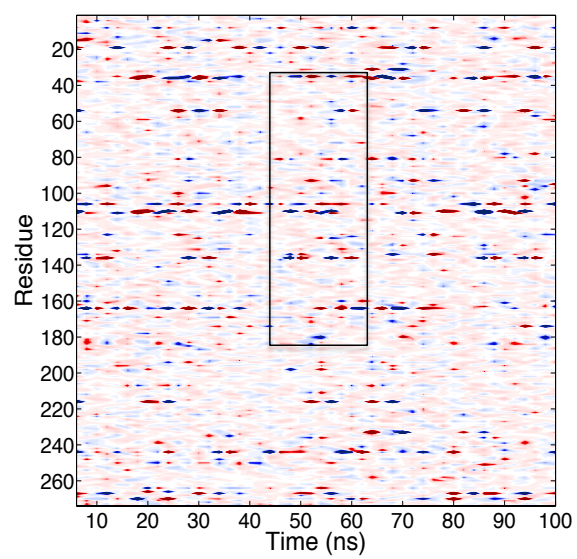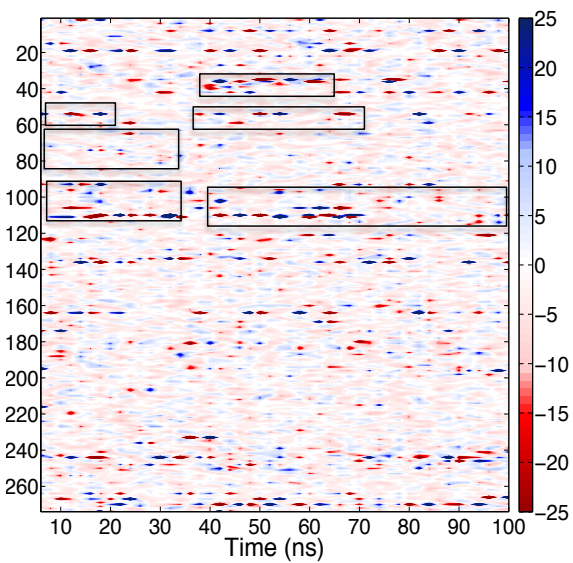

Supplement: Figure S3 — Mechanical coupling variation in subtilisin due to Ca2+ binding. Variation in the force constant of each residue between neighboring time windows for (c) the Ca2+ simulation and (d) the apo simulation. The time window for calculating force constants is 4 ns. (PDF) [file pcbi.1002023.s004.pdf]

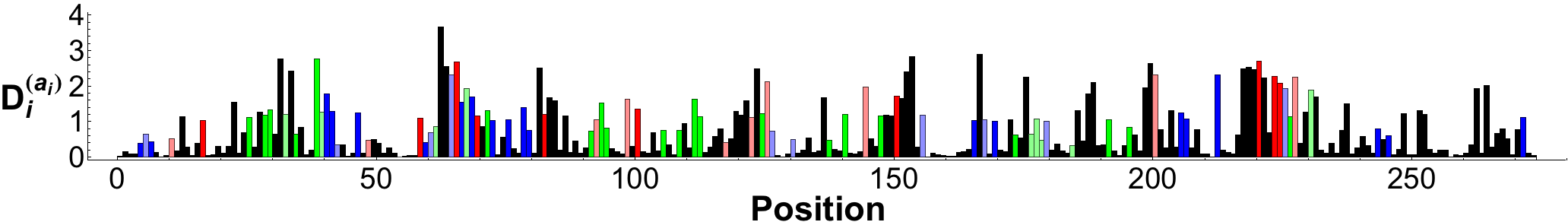

Supplement: Figure S6 — Positional conservation of the multiple sequence alignment, defined as the relative entropy between the observed amino acid frequencies f (a) in each column i and the background frequencies q (a) from all proteins: . Following [33], a binary approximation was applied. Each position is represented as 1 if it contains the most prevalent amino acid in that column, or 0 otherwise. Columns are colored based on the clusters shown in Figure S7. (TIF) [file pcbi.1002023.s007.tif]

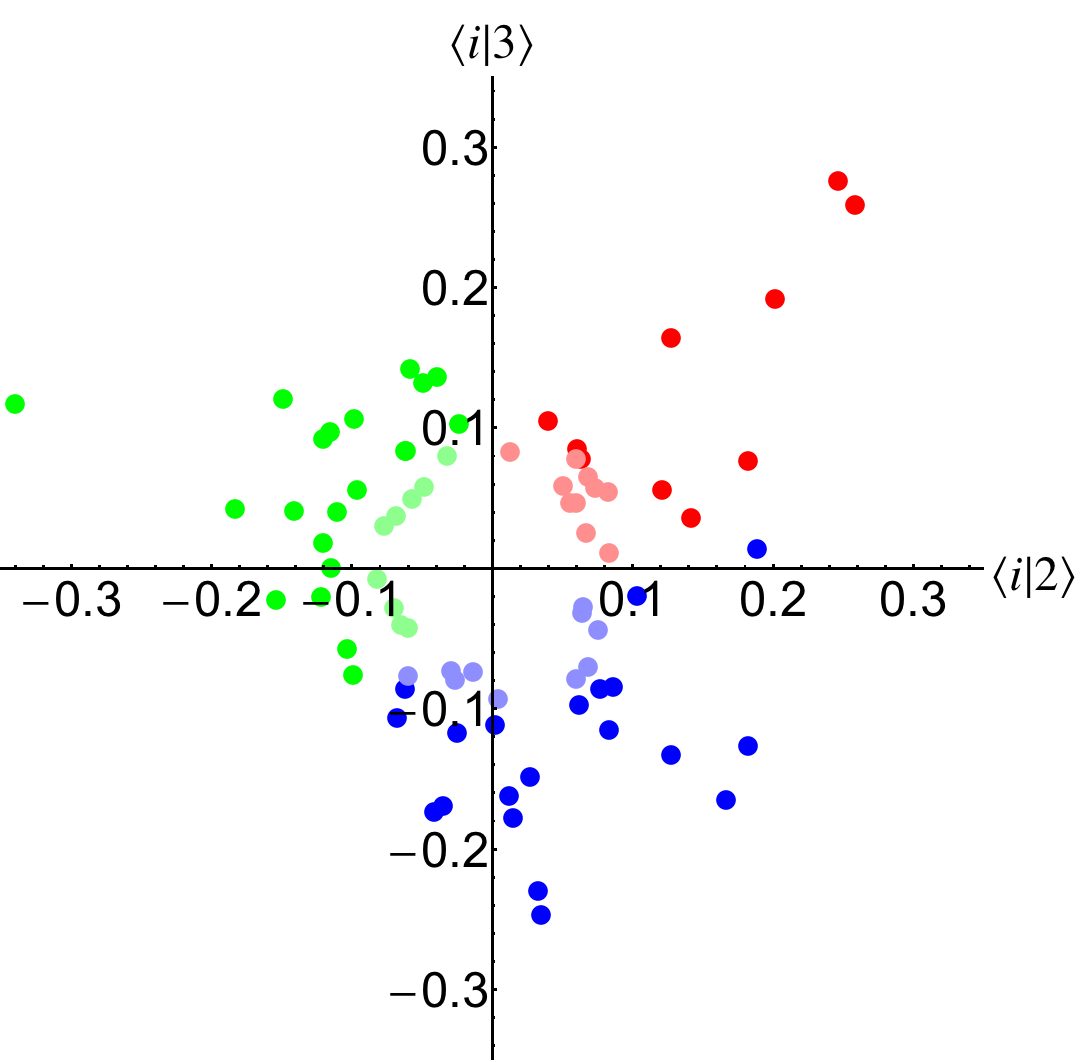

Supplement: Figure S7 — Scatter plot of the 2nd and 3rd eigenvectors. A cutoff distance of 0.07 from the origin was used to select 80 residues that tend to co-evolve, which were divided into three clusters: blue, red, and green. Residues at a distance of 0.07–1.0 from the origin are colored with a lighter shade. (TIF) [file pcbi.1002023.s008.tif]
